# The immeasurable value of plankton to humanity

**DOI:** 10.1093/biosci/biaf049

**Published:** 2025-06-24

**Authors:** Maria Grigoratou, Susanne Menden-Deuer, Abigail McQuatters-Gollop, George Arhonditsis, Luis Felipe Artigas, Sakina-Dorothée Ayata, Dalida Bedikoğlu, Beatrix E Beisner, Bingzhang Chen, Claire Davies, Lillian Diarra, Owoyemi W Elegbeleye, Jason D Everett, Tatiane M Garcia, Wendy C Gentleman, Rodrigo Javier Gonçalves, Tamar Guy-Haim, Svenja Halfter, Jana Hinners, Richard R Horaeb, Jenny A Huggett, Catherine L Johnson, Maria T Kavanaugh, Ana Lara-Lopez, Christian Lindemann, Celeste López-Abbate, Monique Messié, Klas Ove Möller, Enrique Montes, Frank E Muller-Karger, Aimee Neeley, Yusuf Olaleye, Artur P Palacz, Alex J Poulton, A E Friederike Prowe, Lavenia Ratnarajah, Luzmila Rodríguez, Clara Natalia Rodríguez-Flórez, Aurea Rodriquez-Santiago, Cecile S Rousseaux, Juan Francisco Saad, Ioulia Santi, Alice Soccodato, Rowena Stern, Selina Våge, Ioanna Varkitzi, Anthony Richardson

**Affiliations:** Gulf of Maine Research Institute, Portland, Maine, United States; Mercator Ocean International, Toulouse, France; European Polar Board, Umeå, Sweden; Graduate School of Oceanography, University of Rhode Island, Kingston, Rhode Island, United States; School of Biological and Marine Scienceat, University of Plymouth, Plymouth, England, United Kingdom; University of Toronto Scarborough, Toronto, Ontario, Canada; Université du Littoral Côte d'Opale, CNRS, Université de Lille, Lille, France; Sorbonne University, Paris, France; Institute of Marine Sciences and Management, Istanbul University, Istanbul, Turkey; Département des Sciences Biologiques and GRIL, Université du Québec à Montréal, Montréal, Québec, Canada; Department of Mathematics and Statistics, University of Strathclyde, Glasgow, Scotland, United Kingdom; Commonwealth Scientific and Industrial Research Organisation, Canberra, Australian Capital Territory, Australia; Mercator Ocean International, Toulouse, France; Department of Marine Sciences, University of Lagos, Lagos, Nigeria; School of the Environment, Centre for Biodiversity and Conservation Science of the University of Queensland, Brisbane, Queensland; Centre for Marine Science and Innovation, University of New South Wales, Sydney, New South Wales; Commonwealth Scientific and Industrial Research Organization, Environment, Queensland Biosciences Precinct, Brisbane, Queensland, Australia; Universidade Federal do Ceará, Fortaleza, Ceará, Brazil; Department of Engineering Mathematics, Dalhousie University, Halifax, Nova Scotia, Canada; Ecology Department and Modeling Nature Unit, Universidad de Granada, Granada, Andalousia, Spain; Israel Oceanographic and Limnological Research Institute, Department of Life Sciences at Ben-Gurion University of the Negev, Eilat, Israel; National Institute of Water and Atmospheric Research, Auckland, New Zealand; Institute of Carbon Cycles, Helmholtz Zentrum Hereon, Geesthacht, Germany; National Marine Information and Research Centre, Ministry of Fisheries and Marine Resources, Sam Nujoma Marine and Coastal Resources Centre, University of Namibia, Windhoek, Namibia; Department of Forestry, Fisheries, Environment, Ocean and Coasts, Department of Biological Sciences, University of Cape Town, Cape Town, South Africa; Fisheries and Oceans Canada, Bedford Institute of Oceanography, Dartmouth, Nova Scotia, Canada; Oregon State University, Corvallis, Oregon, United States; Institute for Marine and Antarctic Studies, University of Tasmania, Hobart, Tasmania, Australia; Norwegian Institute for Water Research, Department of Biological Sciences, University of Bergen, Bergen, Norway; Instituto Argentino de Oceanografía, Bahía Blanca, Argentina; Monterey Bay Aquarium Research Institute, Monterey, California, United States; Institute of Carbon Cycles, Helmholtz Zentrum Hereon, Geesthacht, Germany; Cooperative Institute for Marine and Atmospheric Studies, Rosenstiel School of Marine, Atmospheric, and Earth Science, University of Miami, Atlantic Oceanographic and Meteorological Laboratory, National Oceanic and Atmospheric Administration, Miami, Florida, United States; College of Marine Science, University of South Florida, Tampa, Florida, United States; NASA, Washington, DC, United States; Department of Marine Sciences, University of Lagos, Lagos, Nigeria; Institute of Oceanology of the Polish Academy of Sciences, Warsaw, Poland; Lyell Centre for Earth and Marine Sciences, Heriot-Watt University, Edinburgh, Scotland, United Kingdom; GEOMAR Helmholtz Centre for Ocean Research Kiel, Kiel, Germany; Australian Antarctic Program Partnership, Institute for Marine and Antarctic Studies, University of Tasmania, Hobart, Tasmania, Australia; Universidad Científica del Sur, Carrera de Biologia Marina, Villa, Peru; Secretariat of Environment and Sustainable Development, Boyacá, Colombia; Taller Ecológico de Puerto Rico, Caribbean Coastal Ocean Observing System, Boquerón, Puerto Rico, United States; Ocean Ecology Laboratory, NASA, Washington, DC, United States; Centro de Investigación Aplicada y Transferencia Tecnológica en Recursos Marinos Almirante Storni, Facultad de Ciencias Marinas, Universidad Nacional del Comahue, In Rio Negro, Argentina; European Marine Biological Resource Centre, Paris, France; Hellenic Center for Marine Research, Institute of Marine Biology Biotechnology and Aquaculture, Crete, Greece; European Marine Biological Resource Centre, Paris, France; Marine Biological Association of the United Kingdom, Plymouth, England, United Kingdom; Department of Biological Sciences, University of Bergen, Bergen, Norway; Hellenic Centre for Marine Research, Crete, Greece; School of the Environment, Centre for Biodiversity and Conservation Science of the University of Queensland, Commonwealth Scientific and Industrial Research Organization, Environment, Brisbane, Queensland, Austalia

**Keywords:** biodiversity, climate, ecology, policy, biogeochemistry

## Abstract

Plankton, a diverse group of aquatic organisms, make Earth livable, regulate aquatic life, and provide benefits to human societies such as access to clean water, food security, and well-being. They also support economies and inspire biotechnological innovations. This article aims to raise awareness of the value of plankton to humanity and serves as an informative guide for aquatic professionals, policymakers, and anyone interested in plankton. We present the value of plankton across six themes of human interest: biogeochemistry; ecology; climate; the evolution of science; economy; and culture, recreation, and well-being. Guided by the 2022 Intergovernmental Science-Policy Platform on Biodiversity and Ecosystem Services values assessment, we introduce the six themes under the Life Framework of Values to offer a comprehensive summary of the significance of plankton to humanity. In addition, we provide examples of plankton variables used in policy frameworks and recommendations for enhancing understanding of their value through long-term sustainable research and monitoring.

Plankton consist of diverse communities suspended in aquatic environments, including thousands of species from all kingdoms (de Vargas et al. 2015, Ruggiero et al. 2015). They exhibit a wide array of shapes and colors, with lifespans of a few hours to more than 5 years (e.g., krill; Nicol 2006). Plankton produce oxygen, store atmospheric carbon, and affect water quality. They support the aquatic ecosystems humans rely on for livelihood and food (Suthers et al. 2019) and are identified as Essential Ocean and Climate Variables (Miloslavich et al. 2018, GCOS 2022). Plankton have a high adaptive capacity that can help buffer against climate-driven changes and external disturbances. However, their distribution, biomass, and traits remain vulnerable to climate change, pollution, and human pressures, potentially affecting ecosystems. Beyond their ecological and biogeochemical importance, plankton research has influenced fields such as medicine, engineering, art, and cultural heritage. Unfortunately, the significant ecological and societal benefits of plankton are often overlooked because of the relative visibility, familiarity, and charisma of larger organisms to the general public and, to some extent, because of the focus on economic losses and restrictions on human aquatic activities caused by some harmful plankton blooms. This can lead to an underappreciation of the essential role of plankton in aquatic ecosystems and life on Earth.

The term *plankton* comes from the ancient Greek word *πλαγκτόν*, meaning “drifter,” which references their difficulty in controlling their horizontal movement against currents. Being such a diverse group, planktonic organisms can obtain their energy from the sun and/or consumption of other organisms, from their host's metabolic processes, and even from inorganic matter (box 1). Plankton have a vast size spectrum (figure 1), from microscopic 0.02-micrometer viruses to some of the world's longest creatures such as the 35-meter-long jellyfish *Cyanea capillata* and 50-meter colonial siphonophore *Praya dubia* (Sardet 2015). However, most plankton are less than a millimeter in size and are invisible to the unaided human eye (figure 1). Despite their small size, plankton exhibit complex behaviors for growth and survival. Some can expand their size or volume (e.g., the freshwater ciliate *Lacrymaria olor*), whereas others release toxins (e.g., the freshwater cyanobacteria *Planktothrix agardhii*, marine dinoflagelates of the *Alexandrium* genus) or build shells and spines (e.g., marine radiolarians and planktonic foraminifera; Vaughn and Allen 2010, Flaum and Prakash 2024). Some have lightning-quick reactions to chemical changes and predators (e.g., dinoflagellates and copepods), and many undertake daily or seasonal vertical migrations (e.g., freshwater and marine copepods, euphausiids, chaetognaths; Takenaka et al. 2017, Bandara et al. 2021, Timsit et al. 2021). Zooplankton vertical migrations span from a few to a thousand meters and are likely the largest animal migrations on Earth in terms of biomass (Hays 2003). A few species can enter a dormancy phase via diapause (e.g., arctic marine copepods of the genera *Calanus* and *Neocalanus*) or the production of resting cysts (e.g., marine diatoms *Thalassiosira*) and eggs (e.g., freshwater and marine cladocerans such as *Daphnia* and *Podon*; box 1). Dormancy helps them to persist through unfavorable conditions (e.g., low temperatures, a lack of sunlight and food in polar regions during winter, starvation, eutrophication, pollution, evaporation in deserts) and recolonize ecosystems once conditions improve (Alekseev and Pinel-Alloul 2019). Many taxa remain planktonic throughout their entire lives (holoplankton), whereas a few others such as fish, sea stars, shellfish, corals, squids, and octopuses have a planktonic stage for only a portion of their lives (meroplankton; box 1, figure 2). Because humans tend to relate to organisms that they can directly observe, hear, taste, smell, or touch, it can be hard to perceive the magnitude and impact of plankton diversity in space and time.

**Figure 1.**
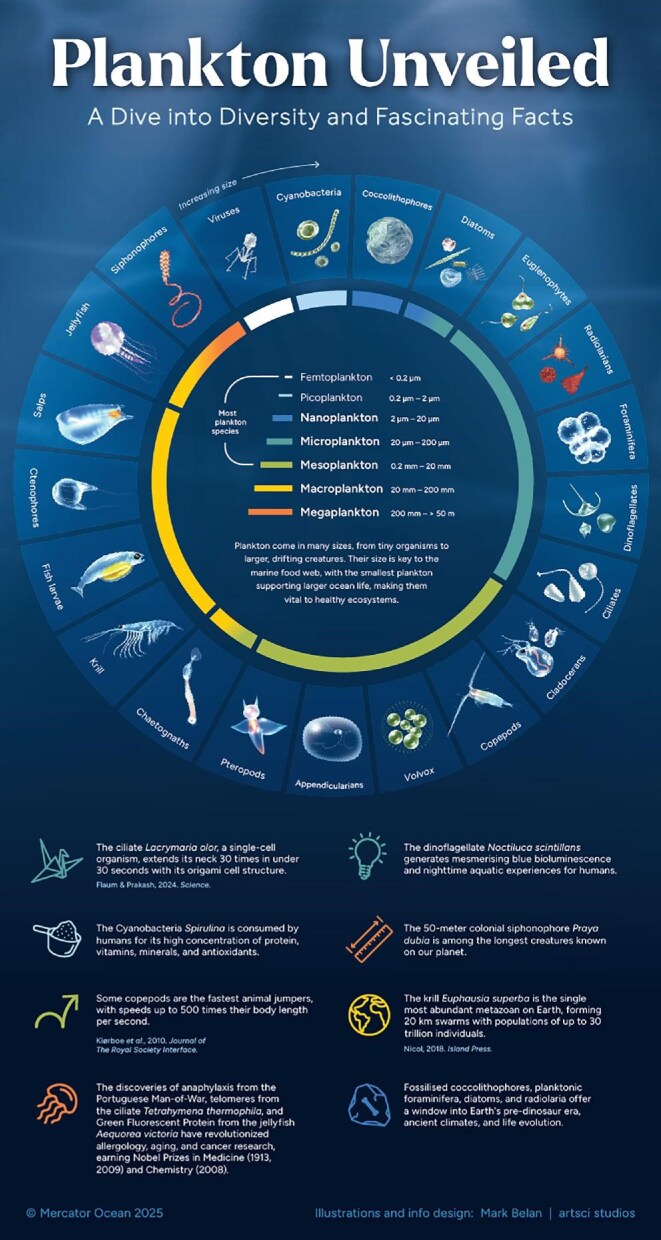
A showcase of plankton size and species diversity, accompanied by fascinating facts highlighting their significance in research and impact on Earth and humanity.

**Figure 2.**
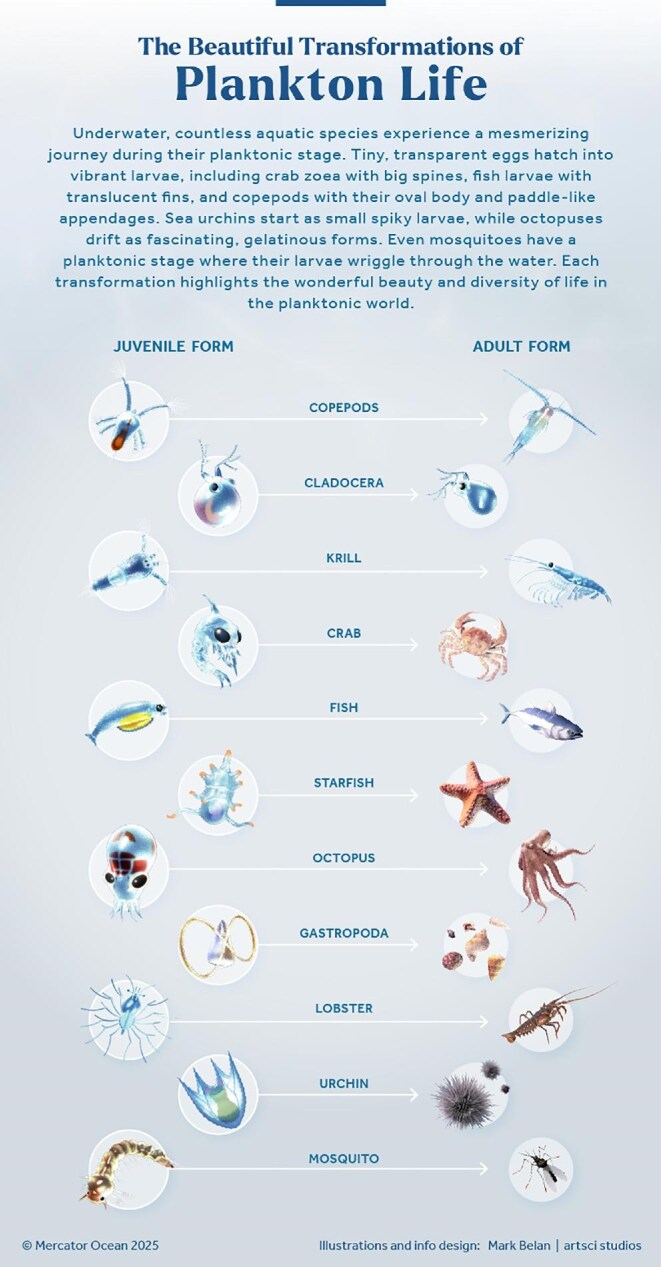
Morphological transformations that occur in holoplanktonic and meroplanktonic organisms during their development from juvenile to adult forms.

Box 1. **Glossary of plankton terminologies mentioned in the article.**

**Detritivores:** Organisms that consume detritus or decomposing organic matter.

**Diapause:** A physiological state of low metabolic activity that allows some plankton species to survive during seasonal unfavorable environmental conditions (e.g., low temperature, nutrient depletion) at the cost of suspended development and reproduction.

**Eukaryotes:** Organisms with a nucleus and other membrane-bound organelles in their cells.

**Holoplankton:** Organisms that spend their entire life cycle as plankton.

**Meroplankton:** Organisms that have a planktonic stage in their life cycle.

**Metazoans (planktonic):** multicellular zooplankton (as opposed to protozoans, single-cell zooplankton).

**Microplankton:** Planktonic organisms between 20 and 200 μm in size.

**Mixoplankton:** Planktonic organisms that can obtain their energy using a mixture of phagotrophy and photosynthesis.

**Phagotrophy:** feeding mechanism by which an organism can consume other organisms or particles by ingesting and internalizing them within its cells

**Phytoplankton:** traditionally, photosynthetic planktonic organisms (microalgae and cyanobacteria). Nowadays it is considered that many of these species are actually mixoplankton.

**Plankton vertical migration:** the upward and downward active movement of plankton in the water column. This daily or seasonal movement is influenced by environmental factors (e.g., light, nutrient availability, predation) and plays a significant role in biogeochemical cycles.

**Plankton:** a diverse group of aquatic organisms (thousands of species from all kingdoms) that live suspended in the water column, whose horizontal distribution is mostly dictated by water currents.

**Prokaryotes:** Single-celled organisms that lack a nucleus and membrane-bound organelles.

**Resting cysts or eggs:** The planktonic resting cysts and eggs have thick outer layers that allow them to persist in sediments during harsh environmental conditions or seasonal changes. They can stay in a dormant stage for weeks to decades. They hatch when the environmental conditions become favorable for the organism to resume growth and reproduction.

**Zooplankton:** Planktonic heterotrophic organisms that gain energy and nutrients through the consumption of other organisms or organic sources (e.g., detritus, decomposing organic matter).

This Viewpoint aims to raise awareness and provide a comprehensive understanding of the value of plankton to humanity. It covers all aquatic environments and plankton groups with an emphasis on marine phytoplankton, mixoplankton, and zooplankton (box 1). We highlight the value of plankton in the context of six broad themes of human interest: biogeochemistry; ecology; climate; the evolution of science; economy; and culture, recreation, and well-being. We provide examples of plankton variables used in national and international policy frameworks and propose key priorities for enhancing plankton research to advance our understanding of the mechanisms and functionality of plankton. Guided by the 2022 Intergovernmental Science-Policy Platform on Biodiversity and Ecosystem Services’ (IPBES) values assessment, we introduce the six themes of the value of plankton under the Life Framework of Values (living with, from, in, and as nature; figure 3) by which we aim to offer a comprehensive view of the human–nature interactions, addressing coexistence, resource use, and interconnectedness (O'Neill 1992, O'Connor and Kenter 2019). This article is intended for aquatic ecosystem professionals, policymakers, plankton enthusiasts, and anyone curious about this extraordinary realm of life.

**Figure 3.**
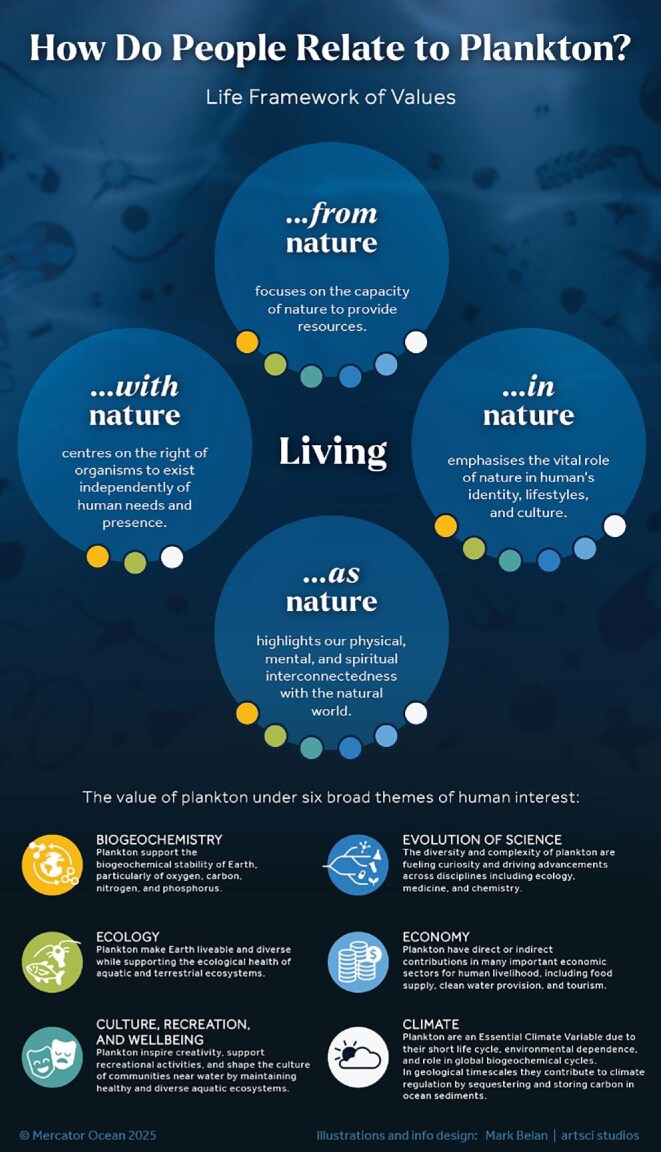
An illustrative overview of the significance of plankton to humanity, contextualised within the Life Framework of Values. The value of plankton is presented across six broad themes of human interest: biogeochemistry; ecology; culture, recreation, and well-being; the evolution of science; economy; and climate.

## The value of plankton

### 1. Plankton and ecology

**Figure 4.**
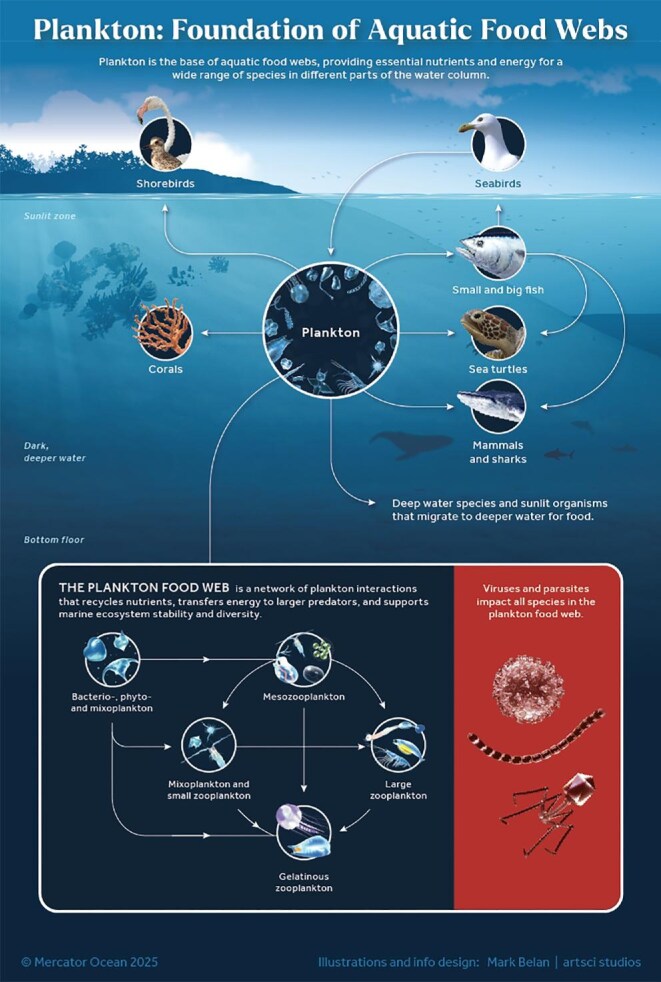
A graphical illustration highlighting the essential role of plankton as the foundation of aquatic food webs.

Plankton have a vital role in sustaining and regulating life in aquatic environments by influencing nutrition, food webs, organism dispersal, and bioinvasion. Fueled by the sun in sunlit ecosystems, phytoplankton transfer energy throughout the water column and sediments via sinking and to higher predators via the food web (figure 4; Siegel et al. 2023). Planktonic primary producers can also synthesize *de novo* n-3 polyunsaturated fatty acids, which can be critical for maintaining high growth, survival, and reproductive rates and for realizing high food conversion efficiencies for a wide range of freshwater and marine organisms (Perhar et al. 2012).

Plankton species play a vital role in nutrient transfer through bioaccumulation, supporting ecosystems by moving essential nutrients through the food web. However, they can also absorb and pass harmful substances, such as pollutants and toxins, which may affect the health and lifespan of species that consume them (Ravera 2001).

Many aquatic animals begin their life cycles as meroplankton (box 1), preying on their fellow plankton for growth while undergoing remarkable transformations (figure 2). Even nonplanktonic species (e.g., sharks, mammals, reptiles) depend on plankton directly or indirectly for their prey. For example, planktivorous fishes (e.g., sardines, hilsa) are key prey for larger predators such as fish (e.g., trout, bass, salmon, tuna, marlin, which represent an important source of food for humans), birds (e.g., puffins), and mammals (e.g., whales; Carpenter et al. 2001, Kotterba et al. 2024). Plankton blooms in surface waters serve as important feeding hotspots for migratory and nonmigratory species and contribute to the overall productivity and biodiversity in both marine and freshwater ecosystems (Behrenfeld and Boss 2014, Huisman et al. 2018). However, some plankton blooms, particularly excessive ones, can have negative effects on the environment, leading to toxic blooms, mass mortality events, and, in some cases, deoxygenation, all of which can occur before the blooms collapse. (García-Mendoza et al. 2018). Bottom-living organisms such as corals and mussels also benefit from the consumption of living plankton and sinking planktonic material. Conversely, excessive plankton concentrations can negatively affect shallow habitats, such as seagrass meadows and coral reefs, by increasing water turbidity and reducing light penetration vital for their survival (Toro-Farmer et al. 2016).

In deep-water habitats where sunlight is absent and photosynthetic plankton are scarce, plankton still influence trophic dynamics and diversity. Through buoyancy and swimming, plankton can adjust their vertical position, facilitating daily or seasonal vertical migrations that span several meters in lakes to thousands of meters in marine environments (Bandara et al. 2021). The vertical migration of living plankton and the passive sinking of marine snow (box 2) transport stored carbon and nutrients from the surface to deeper waters, providing energy for deep-water organisms (figure 4; Turner 2015). These migrations also contribute to the long-term carbon sequestration in the sediments for decades to millennia, helping to mitigate climate change.

Planktonic life stages are also critical for dispersal, enabling organisms to travel long distances via ocean currents, colonize new habitats, and maintain genetic diversity within populations. Human activities such as aquaculture, shipping, and the release of ballast water also transfer plankton species to new environments. This bioinvasion can disrupt food webs when nonnative species outcompete native plankton because of faster growth rates, a lack of natural predators, or other competitive advantages (e.g., Bollens et al. 2002). Over the past six decades, planktonic invasions have resulted in an estimated global economic impact of approximately US$5.8 billion, largely driven by the spread of viruses and invasive zooplankton (Macêdo et al. 2022). Therefore, understanding and monitoring plankton ecology is essential to anticipate potential impacts on biodiversity, human health, and welfare.

### 2. Plankton and biogeochemistry

Photosynthesis, the process of converting light energy to chemical energy, is a fundamentally important chemical reaction to life as it has evolved on Earth. It powers much of Earth's life and produces the oxygen that is critical for the survival of many species on Earth. In aquatic ecosystems, photosynthesis provides the energetic basis to produce organic matter in most food webs that sustains aquatic organisms, from microbes to top-level predators. As photosynthesis is light dependent, it is restricted to sunlit surface waters where a myriad of mostly microbial photosynthetic pro- and eukaryotic species, or phytoplankton (box 1), use diverse pigments and physiological pathways to generate organic matter that feeds higher trophic levels (Falkowski 2002). Note that although thousands of phytoplankton species have been described, the discovery of new species continues (de Vargas et al. 2015).

The amount and flow of energy in marine ecosystems from tiny photosynthetic plankton to top predators is the key to many global processes, including fisheries production and cycles of carbon, nitrogen, phosphorous, silica and other, often limiting, elements. Plankton have an immense biogeochemical footprint because of their roles as producers, consumers, and recyclers in waters globally. Most, if not all, global elemental cycles are facilitated in key steps by microbes, including plankton (Falkowski et al. 2008). Plankton transform and use elements in specific ratios that reflect requirements for building carbohydrates, lipids, proteins, and other building blocks of life (Moreno and Martiny 2018). Nutrients can be limiting in ways that affect global patterns of plankton biodiversity. Some species thrive in the low-latitude Atlantic Ocean because the deposition of Saharan dust delivers iron that would otherwise limit photosynthesis (Mills et al. 2004), whereas requirements by other plankton result in the Great Calcite Belt of the Southern Ocean (Balch et al. 2016). The global biogeochemical footprint of plankton also extends to the atmosphere. Plankton produce dimethyl sulfide that enters the atmosphere and affects cloud formation and climate regulation. Fossilized diatoms from paleolakes drift as airborne particles during massive Saharan dust storms, and are carried over from Africa to South America thanks to the trade winds, fertilizing the Amazon rainforests and the equatorial Atlantic Ocean with iron minerals (Barkley et al. 2021).

**Figure 5.**
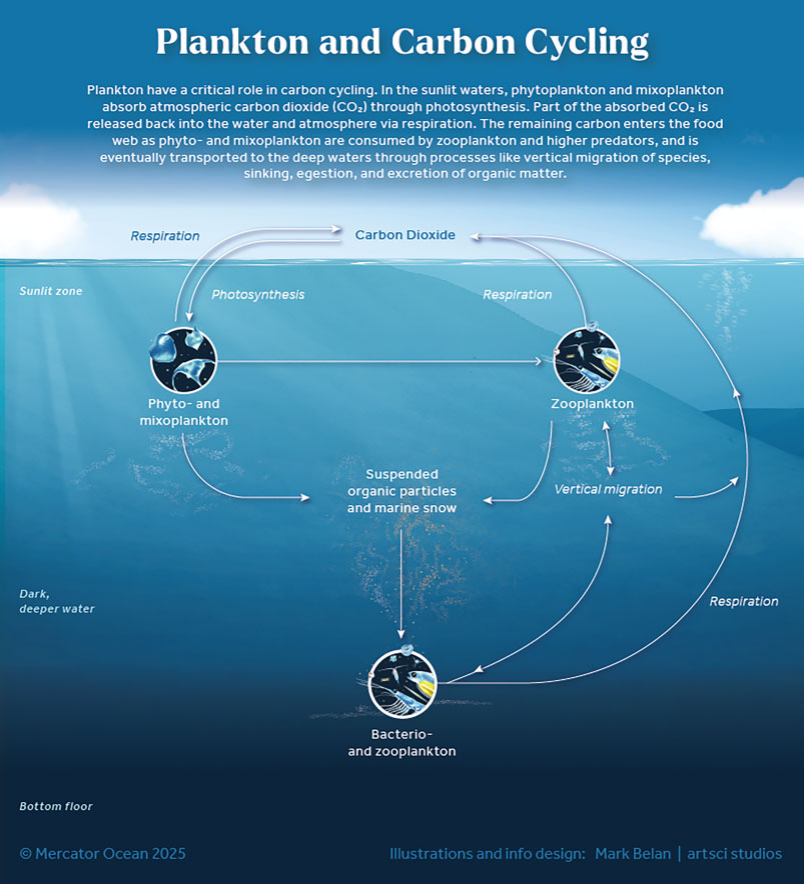
A schematic diagram illustrating the intricate influence of plankton on carbon cycling within aquatic ecosystems.

Global elemental cycles are linked through planktonic metabolism and ecological interactions that move nutrients through the biosphere; that is, elements are moved by the processes that make up life and death (Steinberg and Landry 2017, Tanioka et al. 2022). Atmospheric concentrations of major elements are regulated by surface ocean production and subsequent export of organic matter to ocean depths. A vertical gradient in major elements is maintained via biologically mediated and enhanced transport through vertical migration, sinking, egestion, and excretion of organic matter, a process collectively called the *biological pump* (figure 5, box 2; Siegel et al. 2023). Over time, a scientific consensus has emerged that resolves discrepancies in fossil fuel-derived carbon dioxide emissions by identifying the ocean as a significant absorber of the excess carbon dioxide released into the atmosphere. By some estimates, current atmospheric carbon dioxide concentrations could be up twice as high without this flux mediated by plankton (Friedlingstein et al. 2022). The biological pump is but one example of how important microscopic plankton are in mediating global biogeochemical processes. Therefore, plankton clearly have a remarkable role in making Earth habitable for humans. Their ecology and diversity, yet to be fully understood, are as complex and fascinating as examples from the macroscopic world.

Box 2. **Glossary of terminologies related to aquatic ecosystems mentioned in the article.**

**Acidification:** The process of becoming more acidic which can occur in all aquatic ecosystems. Acidification is often used in reference to a decline in the pH of the ocean owing to the absorption of excess atmospheric carbon dioxide, which can impact marine life, especially calcifying organisms.

**Bioaccumulation:** The accumulation of substances, such as toxins or pollutants, within an organism over time.

**Biological pump:** A set of interconnected processes that result in the net transport of atmospheric carbon from surface waters to the ocean interior/sediments. It is driven by the activities of marine organisms, and includes the fixation of carbon by aquatic plants, phytoplankton and mixoplankton through photosynthesis, the release of carbon dioxide via respiration, the storage of carbon by animals via prey consumption, and the vertical grantient of organic matter from sunlit to deeper layers of the water colum through vertical migration, sinking, egestion, and excretion. The biological carbon pump has a critical role in regulating Earth's climate, maintaining ocean chemistry, and supporting the productivity and resilience of marine Ecosystems.

**Bioluminescence:** The emission of light by living organisms, such as some planktonic species, through a series of chemical reactions, often used for communication, prey attraction, or predator defence.

**Brownification:** The darkening of surface aquatic waters, usually lakes, fjords and coastal areas due to the increased input of terrestrial organic matter.

**Calcification:** The formation of calcite or aragonite shells and/or spines in many aquatic species, including plankton (e.g., coccolithophores, planktonic foraminifera).

**Eutrophication:** A perturbation process of some aquatic ecosystems that includes a rapid growth of planktonic autotrophs, fuelled by an increased availability nitrogen and phosphorus, along with high temperatures. This process can occur naturally or be caused/accelerated by human activities, such as agricultural runoff and global warming. When nutrient levels become excessive, eutrophication can degrade water quality, leading to hypoxia (oxygen depletion) and the death of various aquatic species.

**Marine snow:** A continuous shower of organic and inorganic material (e.g., CaCO3, opal), including dead and decaying plankton, faecal pellets, and other debris, that sinks from sunlit to deeper waters. Marine snow ends up as food for different organisms (while it sinks through the water column) or it reaches the seafloor where it may remain ‘sequestered’ for thousands of years.

### 3. Plankton and climate

Plankton have been recognized as an Essential Climate Variable (GCOS 2022) because of their short lifespan, strong reliance on the physical properties of their habitats, and critical role in the global carbon cycle and other biogeochemical processes (for more details, see the “Plankton and biogeochemistry” section; Hays et al. 2005). Over geological timescales, plankton play a significant role in climate regulation through carbon capture via photosynthesis and export via the biological pump (box 2), ultimately leading to long-term carbon sequestration in ocean sediments (Siegel et al. 2023). Modern and fossilized species, including resting cysts and eggs (box 1), allow scientists to reconstruct Earth's climate history and evaluate the effects of climate change on ecosystems through time (Gray et al. 2012, Trubovitz et al. 2020, Benedetti et al. 2021).

**Figure 6.**
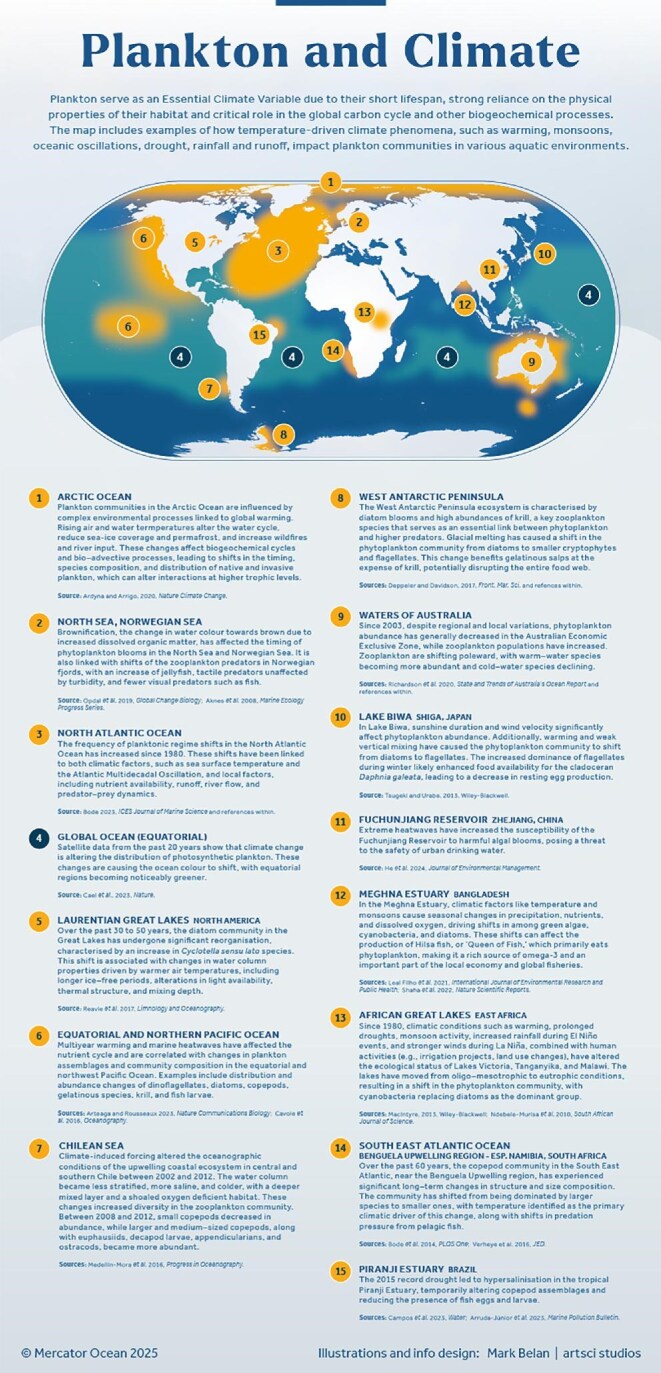
A map with local, regional, and global examples where temperature-driven climate phenomena, such as monsoons, oceanic oscillations, stratification, drought, and hypersalination, have affected plankton communities across various aquatic systems, including lakes, estuaries, and coastal and open ocean environments.

Temperature is one of the main climate factors that influences various aspects of plankton life, including metabolism, growth, reproduction, morphology, and survival rates (Zohary et al. 2021, Ratnarajah et al. 2023). Warming can also affect plankton indirectly via changes in the water cycle because ocean circulation, precipitation patterns, sea ice dynamics, and water column stratification lead to changes in nutrient and light availability needed for plankton growth (figure 6; Winder and Sommer 2012, Woolway et al. 2020). For example, drought conditions can reduce water availability and habitat connectivity in freshwater and estuarine ecosystem-fragmenting plankton populations and limiting dispersal (Rojo et al. 2012, Campos et al. 2022). On the other hand, rainfall and runoff may increase terrestrial input and nutrient concentrations and contribute to a brownification effect in lakes and coastal regions (box 2). This phenomenon may provide zooplankton with protection against ultraviolet radiation (Wolf and Heuschele 2018) and visual predators such as fish (Jönsson et al. 2011) but also may cause shifts in phytoplankton composition, concentration, and blooms (Opdal et al. 2019). Reduced ice coverage in polar habitats influences light penetration and nutrient cycling, affecting the phenology (i.e., life-cycle timing), functionality, and the blooms of various plankton species (Deppeler and Davidson 2017, Ardyna and Arrigo 2020). Aquatic acidification (box 2) challenges the ability of calcifying plankton such as foraminifera, coccolithophores, pteropods, and the larvae of echinoderms and mollusks to form and maintain shells, potentially affecting their role in carbon cycling and marine food webs (Tyrrell 2008, Martins Medeiros and Souza 2023). Observations and modeling studies have shown that ongoing climate change has altered the distribution of plankton in aquatic ecosystems. For example, 20 years of satellite data have recorded a color change in the ocean with equatorial regions becoming noticeably greener because of the distribution shifts of photosynthetic plankton species (figure 6; Cael et al. 2023).

Field observations have also demonstrated changes in the distribution of marine plankton populations including poleward shifts (Poloczanska et al. 2013). These trends are expected to continue, with models predicting additional distribution changes and plankton biomass declines by 2100 (Benedetti et al. 2021, Cooley et al. 2022, Heneghan et al. 2024). Warming and stratification caused by climate change are also connected to plankton population changes in freshwater alpine, temperate and tropical lakes (Shimoda et al. 2011, Michelutti et al. 2015, Ogutu-Ohwayo et al. 2016).

The consequences of climate-driven changes in plankton communities extend far beyond the plankton themselves. They can significantly affect both marine and terrestrial ecosystems, affecting key processes such as nutrient cycling, carbon sequestration, and food web dynamics (Hays et al. 2005). Therefore, studying the plankton responses to climate change is critical in understanding, predicting, and addressing the broader implications of climate change on Earth and the well-being of our societies.

### 4. Plankton and the evolution of science

From the intricate structures and astonishing colors to the remarkable adaptations of single-celled organisms and the complex life cycles of planktonic metazoans (box 1), human curiosity and amusement have been fueled by plankton. Repeatedly, knowledge gained by studying plankton has been applied to many other organisms and ecosystems in water and on land. Plankton research has advanced ecological concepts such as the paradox of plankton (Hutchinson 1961) and competition theory (Tilman 1982), with plankton-driven models enhancing ecosystem understanding, hypothesis testing, and experimental design. Plankton data inspired the hypothesis of equal biomass distribution in logarithmic size classes, forming the basis of size-spectrum theory (Platt and Denman 1977), validated across diverse organisms (Hatton et al. 2022). Studying plankton continues to yield insights into allometry and scaling relationships between size and biological traits.

Our understanding of the diversity and limits of life is broadened by plankton. Extremophilic plankton thriving in harsh environments (e.g., deep-sea hydrothermal vents, polar regions) provide insights into adaptability, evolution, and extraterrestrial habitability (Calbet 2024). Fossilized plankton have advanced paleoceanography and the reconstruction of Earth's history, revealing climate changes and life evolution (Rigby and Milsom 2000, Falkowski et al. 2004). Plankton colonies (e.g., *Volvox*, choanoflagellates, siphonophores) enhance our understanding of multicellularity and individuality in nature (Miller 2010), and the chordate bodies of salps and larvaceans help us study vertebrate and human evolutionary processes.

Fields beyond biological sciences, such as physics, mathematics, medicine, socioeconomics, forensics, engineering, and citizen science have been inspired by planktonic organisms. The competitive exclusion principle derived from plankton has applications in socioeconomics (Gause 1934), and plankton species have been used as model organisms to study nonlocal reaction–diffusion equations in mathematics (e.g., Du and Hsu 2010). Plankton-inspired research has developed theories and tools for understanding concepts such as buoyancy and gravity underwater (Kiørboe et al. 2018, Krishnamurthy et al. 2019). Mixoplankton enables research in phagotrophy (box 1) and endosymbiotic plastid acquisition (Millette et al. 2023) with potential implications for organ transplants. The notable discoveries of anaphylaxis from the Portuguese man o’ war (*Physalia physalis*), the telomeres from the freshwater ciliate *Tetrahymena thermophila*, and the green fluorescent protein from the jellyfish *Aequorea victoria* have revolutionized allergology, ageing, and cancer research, earning Nobel Prizes in medicine (1913, 2009) and chemistry (2008; Blackburn 2010, Botterell et al. 2023). Freshwater plankton species such as rotifers and cladocerans (e.g., *Moina macrocopa, Daphnia magna*) are used as role models in biomedical and ecotoxicological research (Dahms et al. 2011, Siciliano et al. 2015). In forensic science, diatoms are used as diagnostic tools for determining deaths by drowning (Saini and Rohilla 2020).

Phytoplankton communities are so dense in the sunlit waters that they can be seen from space. The launch of the Coastal Zone Color Scanner by NASA in 1979 proved the concept that phytoplankton biomass can be estimated from space by measuring an important trait of these organisms: color. This advancement in space-based plankton observation has greatly influenced ecology and led to the development of numerous satellite and airborne sensors from space agencies worldwide. The new generation of satellite sensors will be able to measure even more colors to help us better understand the diversity of phytoplankton functional groups in aquatic systems. Moreover, plankton-inspired frugal science aims to develop low-cost, high-quality tools to democratize science access (de Vargas et al. 2022). Frugal science has already led to groundbreaking discoveries related to the rapid expansion of plankton size for survival, including cellular origami techniques (Flaum and Prakash 2024) and hydrodynamic trigger waves (Mathijssen et al. 2019). Citizen science and outreach activities provide unique opportunities for individuals of all ages to engage in scientific research, enhance their knowledge of plankton and aquatic ecosystems, and inspire future generations of scientists (supplemental table S1; Kirby et al. 2021).

### 5. Plankton and the economy

Plankton are an often underestimated part of various economic sectors, including food supply, access to water, tourism, energy supply, and biotechnology. Plankton fisheries harvest jellyfish, krill, and copepods. The most common cultured plankton organisms are the cyanobacterium *Spirulina*, the shrimp *Artemia*, and rotifers of the genus *Brachionus* (Suthers et al. 2019, Araujo et al. 2022). Harvested and farmed plankton are used as food and supplements for species cultured for both commercial and recreational aquaculture, such as fish, shellfish, and shrimp, as well as for humans (e.g., krill quesadillas, Calanus soups, jelly dishes, Spirulina powder, plankton oils, polyunsaturated fatty acids). As the regulator of aquatic life, plankton also affect the populations and distributions of many organisms with socioeconomic importance. Upwelling systems worldwide, such as those off the coasts of Peru, West Africa, Western North America, and Venezuela, demonstrate the economic significance of plankton. The Peruvian upwelling system, for instance, is an example of how nutrient-rich waters from ocean depths fuel high productivity and plankton growth which in turn sustains nearly 10% of the global fish catch (Chavez et al. 2008). Some countries, however, might see economic losses when environmental conditions trigger blooms of harmful algae or jellyfish (Richardson et al. 2009, Griffith and Gobler 2020). As an example, the 2017–2019 Red Tide event in Southwest Florida resulted in over US$184 million in local monetary losses and nearly 3000 job-years lost (Court et al. 2021). Open and controlled aquacultures can also be affected by unregulated plankton blooms, which may deplete oxygen levels and elevate concentrations of toxins and parasites, thereby threatening the health of cultured species and posing risks to their consumers.

Plankton support economic sectors beyond food supply. By acting as natural biofilters, some plankton contribute to the removal of excess nutrients and pollutants from water bodies. This contributes to clean water provision and benefits sectors related to human water consumption and use, agriculture, and industrial manufacturing and cooling. Plankton populations also help maintain diverse aquatic habitats, promoting recreational activities that generate substantial revenue for industry and employment opportunities in communities close to water.

In addition to these benefits, plankton also have a broader economic impact through their exploitation in various sectors such as medicine, cosmetics, construction, and energy supply. For example, the freshwater mixoplankton *Haematococcus pluvialis* is farmed for its astaxanthin, which is widely used in pharmaceuticals, cosmetics, and food colorants (e.g., salmon, Régnier et al. 2015). Plankton-derived bioactive compounds, such as bioluminescent proteins and toxins, are increasingly used in medicine and the pharmaceutical industry. Applications include nonpolluting fluorescent markers and products with therapeutic potential, such as antibiotics, antivirals, anticancer agents, and immunomodulatory drugs (Abida et al. 2013, Riccio and Lauritano 2019). Estimates indicate that marine bacteria may account for up to 64% of the US$563 billion to US$5.69 trillion market value in undiscovered marine-derived anticancer drugs (Erwin et al. 2010). Researchers have also explored the use of selected plankton algae as more environmentally friendly alternatives to pesticides for controlling the planktonic stages of vectorial mosquitoes through toxicity or indigestibility (Marten 2007). Calcite shells of certain plankton species (e.g., foraminifera, coccolithophores) are part of limestone, a material used in the steel industry and for the production of chalk, construction materials, agricultural lime, and toothpaste. Fossil oil and natural gas include thousands on thousands of dead plankton organisms that were buried on the sea floor millions of years ago (Suthers et al. 2019), whereas planktonic microalgae such as *Dunaliella* are used for biodiesel and bioethanol production (Amoozegar et al. 2019, Calbet 2024). Innovative marine carbon dioxide removal strategies, such as ocean alkalinity enhancement and artificial upwelling, are being developed to use plankton in actions toward net-zero emissions by 2050 (Zhang et al. 2022). For successful implementation, these strategies must provide evidence of minimal negative impacts on ocean ecosystems and biodiversity (GESAMP 2019, Zhang et al. 2022).

### 6. Plankton, human culture, recreation, and well-being

Human culture, recreation, and well-being are being supported by plankton in various ways. Communities close to water bodies use plankton as a food source both indirectly, by supporting smaller fish species that contribute to their cultural practices and diets, and directly. For example, in China and Japan, jellyfish feature prominently in traditional dishes as a high nutrition—low calorie culinary delicacy with various health benefits such as aiding digestion and treatment of high blood pressure and bone pain (Leone et al. 2015). The freshwater cyanobacterium *Spirulina* has been part of traditional diets in African communities such as the Kanembu around Lake Chad and Central American communities such as the Aztecs, and its popularity continues to expand globally as a health food supplement.

As key indicators of water quality, plankton modulate access to recreational experiences in aquatic environments such as swimming, surfing, recreational fishing, and underwater exploration. For instance, in high latitudes, recreational activities such as fishing and whale watching are closely tied to the timing of plankton blooms. These blooms create hotspots for marine life and attract migratory and charismatic species that follow them. Authorities and local communities often use plankton blooms to inform decisions about whether or not to engage in aquatic recreational activities or enter aquatic areas. Bioluminescent marine dinoflagellates create stunning displays of light, enhancing nighttime aquatic experiences. They are part of cultural events (e.g., the Redhan lun, “Sea of Stars” phenomenon on Vaadhoo Island in the Maldives) and tourist attractions for many countries. Records of bioluminescent plankton can be found in documentaries, the film *The Beach*, and in many videos and photos online. Fossilized plankton create the chalk landscapes that have attracted people for recreation (e.g., the White Cliffs of Dover, in the United Kingdom) and the creation of huge works of art in the landscape, such as the Uffington White Horse in the United Kingdom.

Throughout history, plankton-derived materials have influenced human societies and cultural heritage. The silica-rich skeletons of diatoms and radiolarians have provided valuable resources, such as flint for tools and weapons during the Stone Age and opal for use in jewelry and religious symbols from civilizations, such as the Mesoamericans, the Arabs, the Romans, and the Greeks (Eckert 1997, Suthers et al. 2019). Today, the structural properties of plankton inspire advancements in architecture, engineering, and biomimetics (Jungck et al. 2019). Architects have been inspired by planktonic forms to design iconic buildings, such as Milan's Galleria Vittorio Emmanuele and the former Monumental Gate (Porte Binet) of Paris. In addition, they have influenced the design of systems for renewable energy technologies such as wind turbines, solar panels, and lightweight cars (Pohl and Nachtigall 2015, Sharma et al. 2021).

**Figure 7.**
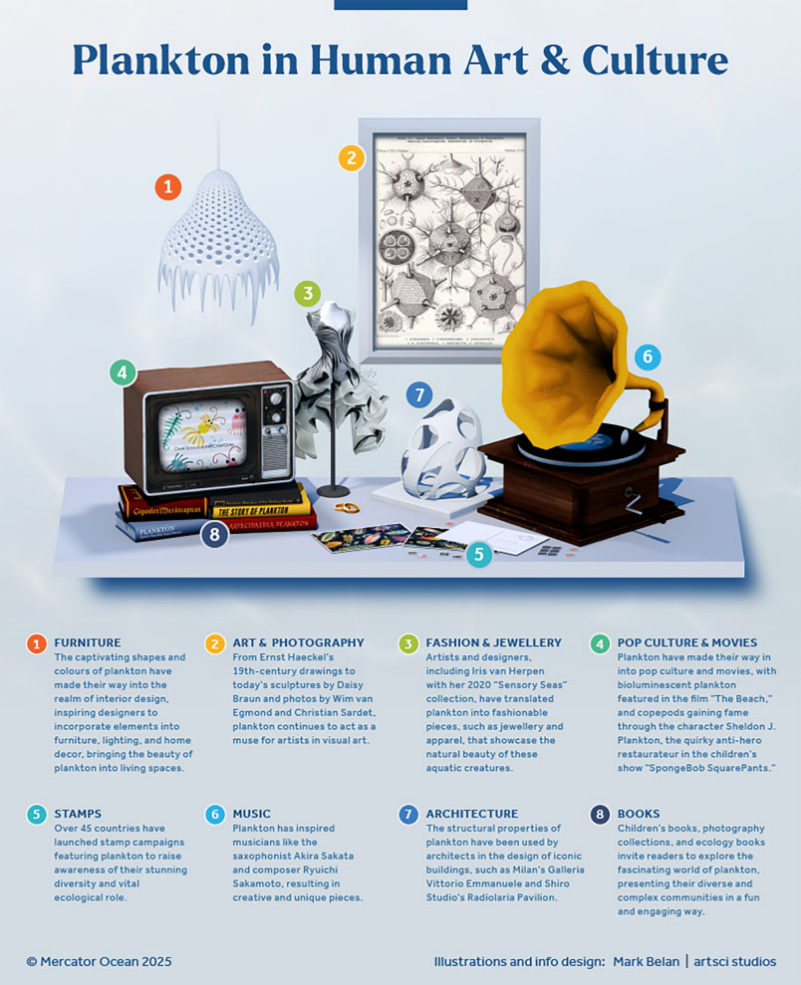
Examples of how plankton organisms have been used as an inspiration in human art and culture.

The intricate forms and vibrant colors of plankton have inspired artists across media, from paintings and sculptures to music, photography, choreography, fashion, and animation (figure 7, supplemental table S2). The detailed plankton illustrations of the nineteenth-century scientist and artist Ernst Haeckel are a remarkable example. The drawings not only introduced the beauty of plankton to a wider audience, they also have inspired many artists over time. Plankton-influenced artworks are displayed in museums, universities, and exhibitions (e.g., 2017 “Wildlife and La Mer” at the Philadelphia Airport), offering an engaging platform for natural history education (Jungck et al. 2019). Plankton have been commemorated on postage stamps globally, with countries raising awareness of their diversity and ecological importance. They have also made their way into popular culture, with characters such as the antihero Sheldon J (a copepod restaurateur) from the children's show *Spongebob Squarepants*. Although this cartoon has helped raise awareness of plankton and although an antihero is not always a negative element in pop culture, its portrayal of plankton as a villain can contribute to a negative perception of plankton among some audiences.

## Plankton and policy

Recent policy initiatives, such as the United Nations Sustainable Development Goal 14 (Life Below Water) and the Kunming–Montreal Global Biodiversity Framework, take a holistic approach to biodiversity management, considering all ecosystem service-supporting species and habitats (Scharlemann et al. 2020). Because of their fundamental role in aquatic ecosystems, plankton biomass and diversity have been identified as Essential Ocean and Climate Variables to be monitored locally in a way that data can be aggregated to evaluate regional and global changes (Miloslavich et al. 2018). Still, despite existing efforts from many nations to monitor plankton communities as indicators of ecosystem health (supplemental table S3), their relevance for ecosystem dynamics and functioning is oftentimes still neither monitored nor assessed. The underrepresentation of plankton in policy mechanisms, discussions on biodiversity loss, and conservation efforts persists, highlighting opportunities to integrate plankton into initiatives such as the Aichi Biodiversity Targets and the Kunming–Montreal Global Biodiversity Framework (Chiba et al. 2018).

The purpose of this policy section is to briefly introduce how the most common plankton variables are used in policy frameworks, with examples from Africa, the Americas, Australia, Europe, and Japan (table S3). Chlorophyll *a* (a variable that reflects phytoplankton biomass) is the most frequently measured variable, followed by primary productivity, community composition (often based on taxonomy and detailed phytoplankton pigment analyses), abundance, and biomass. Some policy frameworks also include monitoring the presence of invasive species (e.g., the International Convention for the Control and Management of Ships’ Ballast Water and Sediments requires the measurement of viable phytoplankton and *Vibrio cholera* in ballast waters) or traits such as size (e.g., the EU Marine Strategy Framework Directive) and toxins (e.g., Australia's Water Quality Improvement Plans).

**Figure 8.**
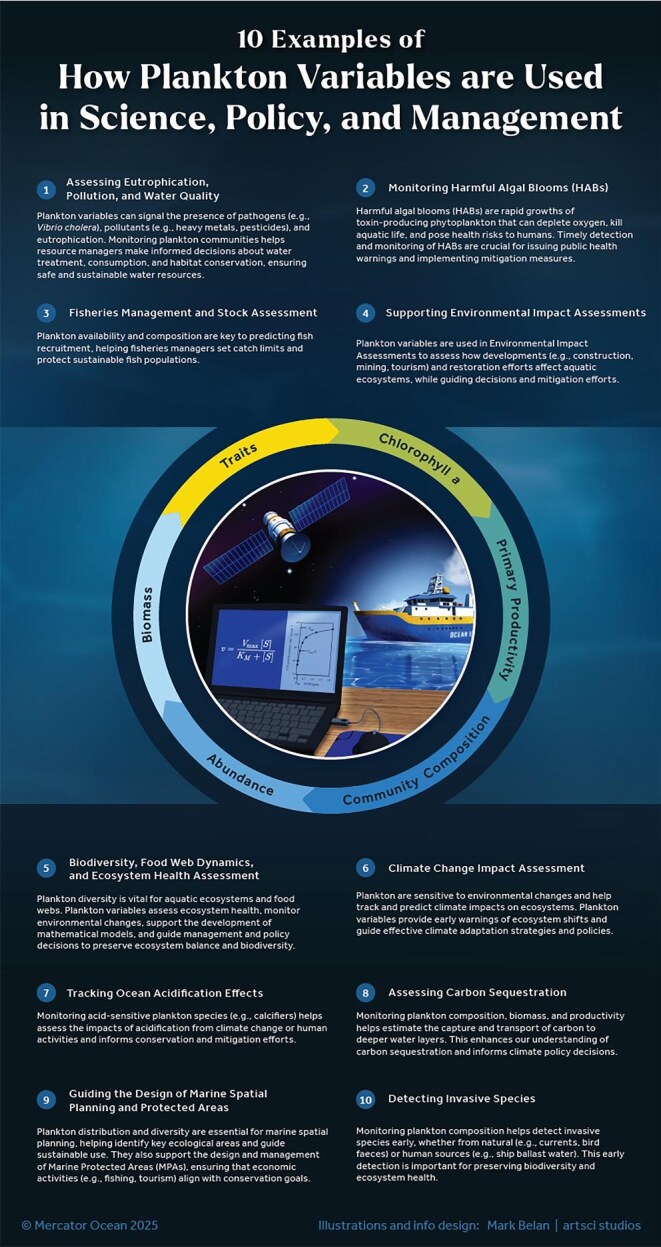
10 examples showcasing the use of common plankton variables (chlorophyll a, primary production, community composition, abundance, biomass, and traits) in scientific research, policy, and environmental management.

Plankton variables serve a wide range of users across legislative mandates (figure 8). They enable government agencies to monitor environmental status and water quality (e.g., microbial pathogens, harmful algae, eutrophication, pollution) and establish protective measures for aquatic ecosystems such as water quality standards (e.g., the Programa de Vigilancia de Playas del Rio, the Periodic Beach Surveillance Programme, of the River Uruguay Administrative Commission; the South African Water Quality Guidelines for Domestic Water Use; the European Water Framework Directive), and marine protected areas (e.g., Canada's Ocean Act, Canada 1996; Japan's Marine Biodiversity Conservation Strategy, Nature Conservation Bureau 2011). Public and private sectors also use plankton variables to monitor the environmental conditions and impacts of their operations (e.g., aquaculture, activities related to tourism), to develop sustainable practices, and to make informed decisions for accessing aquatic ecosystems on the basis of their overall health and quality. Moreover, they are used for monitoring and projecting habitat suitability for the population of fish and charismatic species, ultimately contributing to decisions about sustainable fishing yields. This includes the management of commercially important planktonic species, such as krill (Convention on the Conservation and of Antarctic Marine Living Resources 2023) and the copepod *Calanus finmarchicus* (Nærings og fiskeridepartementet, commercial copepod trawling licenses with total allowable catch).

Despite the use of common plankton variables by various nations, the discrepancies in data collection, analysis, and accessibility both within and among countries pose challenges to the successful implementation of policy frameworks. Establishing a transnational consensus on measurement and analysis standardization, as well as the development of robust methods to compare patterns of change across programs with different sampling methods, would enable more effective data use and facilitate the compilation of information for further research and analysis. In addition, including more ecologically and functionally relevant plankton variables in policy frameworks, such as Essential Biological Variables (Brummitt et al. 2017), would enhance effective ecosystem-based monitoring and forecasting approaches for well-informed decisions on the basis of causality as opposed to just correlational interpretations. This is important, especially when considering disruptive local impacts (e.g., pollution) and climate change on ecosystem stability and the crucial role of plankton in numerous carbon dioxide removal initiatives under development. The creation of an international plankton policy working group has the potential to enhance global awareness and integration of plankton-related issues in high-level policies while still acknowledging that regional policies are important to address specific local needs.

## Essential actions for enhancing our understanding of the value of plankton

Even if plankton have fascinated observers for centuries, it was not until 1887 that Hensen introduced the definition of plankton and that the rise of organized plankton research started (Dolan 2021). Since then, scientists have developed a plethora of tools and methods to study plankton from space, in water, in the laboratory, and with mathematical models (Lombard et al. 2019). In this section, we suggest four key actions to advance and sustain plankton research needed for understanding the values of plankton to humanity and our planet.

### 1. Expanded sustained plankton research

Despite the vital role of plankton observations in ecological studies and policy frameworks, many observing and monitoring programs suffer from underfunding and data accessibility limitations that jeopardize their vital contributions to recording environmental status and understanding aquatic ecosystems (Batten et al. 2019, Ratnarajah et al. 2023). Sustained plankton research requires investments in long-term observing or monitoring programs that measure various plankton groups simultaneously by using different tools (e.g., bottles, nets, omics, imaging, continuous recording systems, optical bulk and single-cell or organism sensors) and can be integrated with satellite and modeling methods (Pierella Karlusich et al. 2022, Ratnarajah et al. 2023). The development of low-cost, high-quality observing tools (e.g., PlanktoScope) and advancements in remote sensing (e.g., satellites, underwater gliders, moorings) can democratize science and expand global data coverage, particularly in undersampled regions across different aquatic environments (Spanbauer et al. 2020). For example, automated technical sensors can be applied on most research vessels, as well of ships of opportunity (e.g., the Continuous Plankton Recorder, FerryBox, GoShip), whereas OneArgo and moorings with imagining tools can collect important information about the biogeography and characteristics of species (Spanbauer et al. 2020, Picheral et al. 2022). Laboratory experiments and mesocosm studies are crucial for understanding plankton ecophysiology. Models consolidate our conceptual understanding and interpolate in time, space, and ecology, making them powerful tools for facilitating hypothesis testing, advancing knowledge, and informing observing systems about critical data needs (Skogen et al. 2021, 2024). For example, plankton digital twins, akin to weather forecasts, can provide projections related to human actions and policy decisions (Flynn et al. 2022). Despite ongoing technological advancements, the continuous investment in educating and supporting plankton experts is vital for sustaining research and enhancing our understanding of the value of plankton to humanity and Earth.

### 2. Harmonized, standardized, and accessible data

Sustainable plankton research can only be guaranteed if observational and modeling data are accessible in almost real time and in a format that can be employed by various users. Ensuring compliance with FAIR (Findable, Accessible, Interoperable, and Reusable) and CARE (Collective Benefit, Authority to Control, Responsibility, and Ethics; Carroll et al. 2021) data principles, along with implementing data standardization protocols such as Darwin Core, significantly enhances interoperability and facilitates seamless data sharing across various platforms (e.g., OBIS, GBIF, COPEPOD). Modeling and forecasting not only synthesize existing data but also generate new data that is important for enhancing our ecological and ecosystem knowledge. Therefore, a standardization of model outputs is needed for increasing their utility. Harmonized data aggregation is key for ensuring accessibility to humans and computational agents that retrieve and integrate diverse data sources for downstream investigations (Wilkinson et al. 2016) and support large-scale research and conservation efforts aligned with international agreements and the UN Sustainable Development Goals.

### 3. Enhancing multidisciplinary collaborations

Multidisciplinary collaborations are crucial for understanding plankton and their ecosystem services. Enhancing cooperation among data providers, scientists, model developers and users is necessary for the optimal use of observational and modeling approaches to forecast plankton as effectively as weather (Lombard et al. 2019, Flynn et al. 2022). Including empiricists in the modeling process from the outset not only enhances model evaluation and calibration but also introduces fresh insights, contextual knowledge, and critical feedback that can refine conceptual models and assumptions. Model developers can offer important directions on field and experimental observational data needs. Effective collaboration ensures that modeling and data collection occur in tandem, leading to more accurate interpretations and practical recommendations that better reflect real-world conditions. In addition, because plankton is part of many social interests (e.g., see the “Plankton and ecology” and “Plankton, human culture, recreation, and well-being” sections), collaborations with experts in socioeconomics, communication, marketing, law, policy, and representatives from Indigenous communities are essential for science-based solutions and conservation efforts, especially in areas at critical risk to anthropogenic activities.

### 4. Advocacy for science education and plankton literacy

By fostering an appreciation for plankton's importance, diversity, and beauty via science education and plankton literacy, we can inspire future generations to sustain planktonic and aquatic ecosystems. Joined actions with artists, citizen scientists, and educators introduce different perspectives and ways of providing observations, communicating, and educating the public (e.g., Garcia-Soto et al. 2017, Garcia et al. 2022). Citizen science and community-driven initiatives offer valuable data and raise awareness. At the same time, outreach efforts that consider local needs and values can deepen community appreciation for plankton and nature, fostering locally tailored sustainable actions (Varanasi et al. 2021).

## Plankton in the Life Framework of Values

The Life Framework of Values (living from, with, in, and as nature) conceptualizes the importance of nature and the ethical responsibilities toward it (figure 3; O'Neill 1992, O'Connor and Kenter 2019). The Intergovernmental Science-Policy Platform on Biodiversity and Ecosystem Services (IPBES) adopts this framework to holistically evaluate the value of nature and help inform policymaking for the sustainable management of biodiversity and ecosystem services (IPBES 2022, Pascual et al. 2023). In the present article, we present how the six themes outlining the value of plankton are integrated within the Life Framework of Values to offer a comprehensive summary of plankton's significance to humanity (figure 3).

*Living from* nature focuses on the capacity of nature to provide resources. Plankton support the biogeochemical stability of aquatic ecosystems, particularly of oxygen, carbon, nitrogen, and phosphorus. By absorbing and storing carbon, plankton contributes to Earth's climate regulation in geological time scales (hundreds to thousands of years). They have a vital role in maintaining aquatic life and high water quality, thereby ensuring global food security and access to water. Plankton deposits are mined for energy (oil, gas, biofuel, flint), agriculture (lime, phosphate), and construction (chalk). Their physiology and ecology contribute to advancements in science including medicine, biotechnology and biomimetics.

*Living with* nature centers on a more harmonious relationship, where humans live in coexistence with nature, respecting its processes and limits including the right of organisms to exist independently of human needs and presence. The myriad of planktonic organisms make Earth livable and diverse by supporting the ecological stability of aquatic ecosystems as well as many terrestrial organisms, and ensuring the continuous link of populations over time and space. A vast number of plankton species’ characteristics, life cycles, and interactions have not yet been described, which puts the focus on this amazing and mysterious realm of life.

*Living in* nature emphasizes the vital role of nature in humans’ identity, lifestyles, and culture. By sustaining healthy and diverse aquatic ecosystems, plankton have a profound impact on culture and recreational activities, especially for communities near water. In addition, plankton inspire creativity in art, literature, and design which fosters a deeper appreciation for these organisms and can promote public awareness about their importance and conservation.

Finally, *living as* nature highlights our physical, mental, and spiritual interconnectedness with the natural world. Recognizing the intrinsic value of plankton not solely as a resource but as an essential part of the Earth's status is pivotal. Cultivating a deep respect for plankton and embracing sustainable practices that ensure their continued abundance and diversity serves as a testament to our commitment to coexist harmoniously with the natural world.

## Supplementary Material

biaf049_Supplemental_File

## Author Biography

Maria Grigoratou (maria.grigoratou1@gmail.com) was affiliated with the Gulf of Maine Research Institute, in Portland, Maine, in the United States, and with Mercator Ocean International, in Toulouse, France, when the manuscript was submitted; she is now affiliated with the European Polar Board, in Umeå, Sweden. Menden-Deuer is affiliated with the Graduate School of Oceanography at the University of Rhode Island, in Kingston, Rhode Island, in the United States. Abigail McQuatters-Gollop is affiliated with the School of Biological and Marine Scienceat the University of Plymouth, in Plymouth, England, in the United Kingdom. George Arhonditsis is affiliated with the University of Toronto Scarborough, in Toronto, Ontario, Canada. Luis Felipe Artigas is affiliated with the Université du Littoral Côte d'Opale, CNRS, at the Université de Lille, in Lille, France. Sakina-Dorothée Ayata is affiliated with the Sorbonne University, in Paris, France. Dalida Bedikoğlu is affiliated with the Institute of Marine Sciences and Management at Istanbul University, in Istanbul, Turkey. Beatrix E. Beisner is affiliated with the Département des Sciences Biologiques and GRIL, at the Université du Québec à Montréal, in Montréal, Québec, Canada. Bingzhang Chen is affiliated with the Department of Mathematics and Statistics at the University of Strathclyde, in Glasgow, Scotland, in the United Kingdom. Claire Davies is affiliated with the Commonwealth Scientific and Industrial Research Organisation, in Canberra, Australian Capital Territory, Australia. Lillian Diarra is affiliated with the Mercator Ocean International, in Toulouse, France. Owoyemi Wahab Elegbeleye is affiliated with the Department of Marine Sciences at the University of Lagos, in Lagos, Nigeria. Jason D. Everett is affiliated with the School of the Environment, Centre for Biodiversity and Conservation Science of the University of Queensland, in Brisbane, Queensland; with the Centre for Marine Science and Innovation at the University of New South Wales, in Sydney, New South Wales; and with the Commonwealth Scientific and Industrial Research Organization, Environment, Queensland Biosciences Precinct, in Brisbane, Queensland, in Australia. Tatiane M. Garcia is affiliated with the Universidade Federal do Ceará, in Fortaleza, Ceará, in Brazil. Wendy C. Gentleman is affiliated with the Department of Engineering Mathematics at Dalhousie University, in Halifax, Nova Scotia, Canada. Rodrigo Javier Gonçalves is affiliated with the Ecology Department and Modeling Nature Unit at the Universidad de Granada, in Granada, Andalousia, Spain. Tamar Guy-Haim is affiliated with the Israel Oceanographic and Limnological Research Institute and with the Department of Life Sciences at Ben-Gurion University of the Negev, in Eilat, Israel. Svenja Halfter is affiliated with the National Institute of Water and Atmospheric Research, in Auckland, New Zealand. Jana Hinners is affiliated with the Institute of Carbon Cycles, Helmholtz Zentrum Hereon, in Geesthacht, Germany. Richard R. Horaeb is affiliated with the National Marine Information and Research Centre, in the Ministry of Fisheries and Marine Resources, and with the the Sam Nujoma Marine and Coastal Resources Centre, at the University of Namibia, in Windhoek, Namibia. Jenny A. Huggett is affiliated with the Department of Forestry, Fisheries, and the Environment, Ocean and Coasts, and with the Department of Biological Sciences at the University of Cape Town, in Cape Town, South Africa. Catherine L. Johnson is affiliated with Fisheries and Oceans Canada, Bedford Institute of Oceanography, in Dartmouth, Nova Scotia, Canada. Maria T. Kavanaugh is affiliated with Oregon State University, in Corvallis, Oregon, in the United States. Ana Lara-Lopez is affiliated with the Institute for Marine and Antarctic Studies at the University of Tasmania, in Hobart, Tasmania, Australia. Christian Lindemann is affiliated with the Norwegian Institute for Water Research and with the Department of Biological Sciences at the University of Bergen, in Bergen, Norway. Celeste López-Abbate is affiliated with the Instituto Argentino de Oceanografía, in Bahía Blanca, Argentina. Monique Messié is affiliated with the Monterey Bay Aquarium Research Institute, in Monterey, California, in the United states. Klas Ove Möller is affiliated with the Institute of Carbon Cycles, Helmholtz Zentrum Hereon, in Geesthacht, Germany. Enrique Montes is affiliated with the Cooperative Institute for Marine and Atmospheric Studies, in the Rosenstiel School of Marine, Atmospheric, and Earth Science, at the University of Miami and with the Atlantic Oceanographic and Meteorological Laboratory, in the National Oceanic and Atmospheric Administration, in Miami, Florida, in the United States. Frank E. Muller-Karger is affiliated with the College of Marine Science at the University of South Florida, in Tampa, Florida, in the United States. Aimee Neeley is affiliated with NASA, in Washington, DC, in the United States. Yusuf Olaleye is affiliated with the Department of Marine Sciences at the University of Lagos, in Lagos, Nigeria. Artur P. Palacz is affiliated with the Institute of Oceanology of the Polish Academy of Sciences, in Warsaw, Poland. Alex J. Poulton is affiliated with the Lyell Centre for Earth and Marine Sciences, at Heriot-Watt University, in Edinburgh, Scotland, in the United Kingdom. A. E. Friederike Prowe is affiliated with GEOMAR Helmholtz Centre for Ocean Research Kiel, in Kiel, Germany. Lavenia Ratnarajah is affiliated with the Australian Antarctic Program Partnership, Institute for Marine and Antarctic Studies, at the University of Tasmania, in Hobart, Tasmania, in Australia. Luzmila Rodríguez is affiliated with the Universidad Científica del Sur, Carrera de Biologia Marina, in Villa, Peru. Clara Natalia Rodríguez-Flórez is affiliated with the Secretariat of Environment and Sustainable Development, in Boyacá, Colombia. Aurea Rodriquez-Santiago is affiliated with the Taller Ecológico de Puerto Rico and with the Caribbean Coastal Ocean Observing System, in Boquerón, Puerto Rico, in the United States. Cecile S. Rousseaux is affiliated with the Ocean Ecology Laboratory at NASA, in Washington, DC, in the United States. Juan Francisco Saad is affiliated with the Centro de Investigación Aplicada y Transferencia Tecnológica en Recursos Marinos Almirante Storni and with the Facultad de Ciencias Marinas at Universidad Nacional del Comahue, In Rio Negro, Argentina. Ioulia Santi is affiliated with the European Marine Biological Resource Centre and with the Hellenic Center for Marine Research at the Institute of Marine Biology Biotechnology and Aquaculture, in Crete, Greece. Alice Soccodato is affiliated with the European Marine Biological Resource Centre, in Paris, France. Rowena Stern is affiliated with the Marine Biological Association of the United Kingdom, in Plymouth, England, in the United Kingdom. Selina Våge is affiliated with the Department of Biological Sciences at the University of Bergen, in Bergen, Norway. Ioanna Varkitzi is affiliated with the Hellenic Centre for Marine Research, in Crete, Greece. Anthony Richardson is affiliated with the School of the Environment and the Centre for Biodiversity and Conservation Science of the University of Queensland and with the Commonwealth Scientific and Industrial Research Organization, Environment, in Brisbane, Queensland, Austalia.

## References

[bib1] Abida H, Ruchaud S, Rios L, Humeau A, Probert I, De Vargas C, Bach S, Bowler C. 2013. Bioprospecting marine plankton. Marine Drugs 11: 4594–4611. 24240981 10.3390/md11114594 PMC3853748

[bib2] Alekseev VR, Pinel-Alloul B. 2019. Dormancy in Aquatic Organisms. Theory, Human Use, and Modeling. Springer International.

[bib3] Amoozegar MA, Safarpour A, Noghabi KA, Bakhtiary T, Ventosa A. 2019. Halophiles and their vast potential in biofuel production. Frontiers in Microbiology 10: 1895. 31507545 10.3389/fmicb.2019.01895 PMC6714587

[bib3a] Araujo GS et al. 2022. Plankton: Environmental and Economic Importance for a Sustainable Future. Plankton Communities. IntechOpen.

[bib4] Ardyna M, Arrigo KR. 2020. Phytoplankton dynamics in a changing Arctic Ocean. Nature Climate Change 10: 892–903.

[bib5] Balch WM et al. 2016. Factors regulating the Great Calcite Belt in the Southern Ocean and its biogeochemical significance. Global Biogeochemical Cycles 30: 1124–1144.

[bib6] Bandara K, Varpe Ø, Wijewardene L, Tverberg V, Eiane K. 2021. Two hundred years of zooplankton vertical migration research. Biological Reviews 96: 1547–1589. 33942990 10.1111/brv.12715

[bib7] Barkley AE, Olson NE, Prospero JM, Gatineau A, Panechou K, Maynard NG, Blackwelder P, China S, Ault AP, Gaston CJ. 2021. Atmospheric transport of North African dust-bearing supermicron freshwater diatoms to South America: Implications for iron transport to the equatorial North Atlantic Ocean. Geophysical Research Letters 48: e2020GL090476.

[bib8] Batten SD et al. 2019. A global plankton diversity monitoring program. Frontiers in Marine Science 6: 321.

[bib9] Behrenfeld MJ, Boss ES. 2014. Resurrecting the ecological underpinnings of Ocean plankton blooms. Annual Review of Marine Science 6: 167–194. 10.1146/annurev-marine-052913-021325 24079309

[bib10] Benedetti F, Vogt M, Elizondo UH, Zimmermann NE, Gruber N, Righetti D. 2021. Major restructuring of marine plankton assemblages under global warming. Nature Communications 12: 5226. 10.1038/s41467-021-25385-x PMC8410869 34471105

[bib11] Blackburn EH. 2010. Telomeres and telomerase: The means to the end (Nobel lecture). Angewandte Chemie 49: 7405–7421. 20821774 10.1002/anie.201002387

[bib11a] Bollens SM, Cordell JR, Avent S, Hooff R. 2002. Zooplankton invasions: a brief review, plus two case studies from the northeast Pacific Ocean. Hydrobiologia 480: 87–110.

[bib12] Botterell ZLR, Lindeque PK, Thompson RC, Beaumont NJ. 2023. An assessment of the ecosystem services of marine zooplankton and the key threats to their provision. Ecosystem Services 63: 101542.

[bib13] Brummitt N, Regan EC, Weatherdon LV, Martin CS, Geijzendorffer IR, Rocchini D, Gavish Y, Haase P, Marsh CJ, Schmeller DS. 2017. Taking stock of nature: Essential biodiversity variables explained. Biological Conservation 213: 252–255.

[bib14] Cael BB, Bisson K, Boss E, Dutkiewicz S, Henson S. 2023. Global climate-change trends detected in indicators of ocean ecology. Nature 619: 551–554. 37438519 10.1038/s41586-023-06321-z PMC10356596

[bib15] Calbet A. 2024. The wonders of Marine Plankton. Pages 95–101 in Calbet A, ed. The Wonders of Marine Plankton. Springer Nature.

[bib16] Campos CC, de Sousa Barroso H, Belmonte G, Rossi S, Soares MO, Garcia TM. 2022. Copepod assemblages at the base of Mangrove food webs during a severe drought. Water 14: 3648.

[bib17] Canada. 1996. Oceans Act (SC 1996, c. 31). Government of Canada.

[bib18] Carpenter SR, Cole JJ, Hodgson JR, Kitchell JF, Pace ML, Bade D, Cottingham KL, Essington TE, Houser JN, Schindler DE. 2001. Trophic cascades, nutrients, and lake productivity: The whole-lake experiments. Ecological Monographs 71: 163–186.

[bib18a] Carroll SR, Herczog E, Hudson M, Russell K, Stall S. 2021. Operationalizing the CARE and FAIR principles for indigenous data futures. Scientific Data 8: 108. 33863927 10.1038/s41597-021-00892-0 PMC8052430

[bib19] Chavez FP, Bertrand A, Guevara-Carrasco R, Soler P, Csirke J. 2008. The northern Humboldt Current System: Brief history, present status and a view towards the future. Progress in Oceanography 79: 95–105.

[bib20] Chiba S, Batten S, Martin CS, Ivory S, Miloslavich P, Weatherdon LV. 2018. Zooplankton monitoring to contribute towards addressing global biodiversity conservation challenges. Journal of Plankton Research 40: 509–518. 30279615 10.1093/plankt/fby030 PMC6159525

[bib21] Convention on the Conservation of Antarctic Marine Living Resources . 2023. Schedule of Conservation Measures 2023/24. Convention on the Conservation of Antarctic Marine Living Resources.

[bib21a] Cooley S et al. 2022. Oceans and coastal ecosystems and their services. In: Climate Change 2022: Impacts, Adaptation and Vulnerability. Contribution of Working Group II to the Sixth Assessment Report of the Intergovernmental Panel on Climate Change, pp. 379–550. Pörtner H-O et al. (eds.). Cambridge: Cambridge University Press and NY: New York, USA.

[bib22] Court C, Ferreira J, Ropicki A, Qiao X, Saha B. 2021. Quantifying the Socio-Economic Impacts of Harmful Algal Blooms in Southwest Florida in 2018. University of Florida Economic Impact Analysis Program.

[bib23] Dahms H-U, Hagiwara A, Lee J-S. 2011. Ecotoxicology, ecophysiology, and mechanistic studies with rotifers. Aquatic Toxicology 101: 1–12. 20961628 10.1016/j.aquatox.2010.09.006

[bib25] Deppeler SL, Davidson AT. 2017. Southern Ocean phytoplankton in a changing climate. Frontiers in Marine Science 4: 40.

[bib26] de Vargas C et al. 2015. Eukaryotic plankton diversity in the sunlit ocean. Science 348: 1261605. 25999516 10.1126/science.1261605

[bib27] de Vargas C et al. 2022. Plankton planet: A frugal, cooperative measure of aquatic life at the planetary scale. Frontiers in Marine Science 9: 936972.

[bib28] Dolan JR. 2021. Pioneers of plankton research: Victor Hensen (1835–1924). Journal of Plankton Research 43: 507–510.

[bib30] Du Y, Hsu S-B. 2010. On a nonlocal reaction-diffusion problem arising from the modeling of phytoplankton growth. SIAM Journal on Mathematical Analysis 42: 1305–1333.

[bib31] Eckert AW. 1997. The World of Opals. New York and Chichester. Wiley.

[bib32] Erwin PM, López-Legentil S, Schuhmann PW. 2010. The pharmaceutical value of marine biodiversity for anti-cancer drug discovery. Ecological Economics 70: 445–451.

[bib36] Falkowski PG. 2002. The ocean's invisible forest. Scientific American 287: 54–61. 10.1038/scientificamerican0802-54 12140954

[bib35] Falkowski PG, Katz ME, Knoll AH, Quigg A, Raven JA, Schofield O, Taylor FJR. 2004. The evolution of modern eukaryotic phytoplankton. Science 305: 354–360. 15256663 10.1126/science.1095964

[bib34] Falkowski PG, Fenchel T, Delong EF. 2008. The microbial engines that drive Earth's biogeochemical cycles. Science 320: 1034–1039. 18497287 10.1126/science.1153213

[bib37] Flaum E, Prakash M. 2024. Curved crease origami and topological singularities enable hyperextensibility of *L. olor*. Science 384: eadk5511. 38843314 10.1126/science.adk5511

[bib38] Flynn KJ, Torres R, Irigoien X, Blackford JC. 2022. Plankton digital twins: A new research tool. Journal of Plankton Research 44: 805–805.

[bib39] Friedlingstein P et al. 2022. Global carbon budget 2022. Earth System Science Data 14: 4811–4900.

[bib40] Garcia TM, Costa ACP, Campos CCC, Júnior JPVA, Barroso H, de S, Soares MdO. 2022. The decade of ocean science: The importance of “rediscovering” the tiny and invisible world of plankton. (A Década da Ciência Oceânica: A importância de “redescobrir” o minúsculo e invisível mundo do plâncton.) Arquivos de Ciências do Mar 55: 102–122.

[bib41] García-Mendoza E, Cáceres-Martínez J, Rivas D, Fimbres-Martinez M, Sánchez-Bravo Y, Vásquez-Yeomans R, Medina-Elizalde J. 2018. Mass mortality of cultivated Northern bluefin tuna *Thunnus thynnus orientalis* associated with *Chattonella* species in Baja California, Mexico. Frontiers in Marine Science 5: 454.

[bib42] Garcia-Soto C et al. 2017. Advancing Citizen Science for Coastal and Ocean Research. European Marine Board. Position paper no. 23.

[bib43] Gause GF. 1934. Experimental analysis of Vito Volterra's mathematical theory of the struggle for existence. Science 79: 16–17. 10.1126/science.79.2036.16-a 17821472

[bib44] [GCOS] Global Climate Observing System . 2022. The 2022 GCOS ECVs Requirements. World Meteorological Organization. GCOS report no. 245.

[bib45] [GESAMP] Joint Group of Experts on the Scientific Aspects of Marine Environmental Protection . 2019. High level review of a wide range of proposed marine geoengineering techniques. GESAMP. Report no. 98.

[bib46] Gray DK, Arnott SE, Shead JA, Derry AM. 2012. The recovery of acid-damaged zooplankton communities in Canadian Lakes: The relative importance of abiotic, biotic and spatial variables. Freshwater Biology 57: 741–758.

[bib47] Griffith AW, Gobler CJ. 2020. Harmful algal blooms: A climate change co-stressor in marine and freshwater ecosystems. Harmful Algae 91: 101590. 32057338 10.1016/j.hal.2019.03.008

[bib48] Grigoratou M et al. 2022. The Marine Biodiversity Observation Network Plankton Workshops: Plankton ecosystem function, biodiversity, and forecasting: Research requirements and applications. Limnology and Oceanography Bulletin 31: 22–26.

[bib49] Hatton I, Heneghan R, Al E. 2022. The global ocean size spectrum from bacteria to whales. Science Advances 7: eabh3732. 10.1126/sciadv.abh3732 PMC8580314 34757796

[bib51] Hays GC. 2003. A review of the adaptive significance and ecosystem consequences of zooplankton diel vertical migrations. Hydrobiologia 503: 163–170.

[bib50] Hays G, Richardson A, Robinson C. 2005. Climate change and marine plankton. Trends in Ecology and Evolution 20: 337–344. 16701390 10.1016/j.tree.2005.03.004

[bib52] Heneghan RF, Holloway-Brown J, Gasol JM, Herndl GJ, Morán XAG, Galbraith ED. 2024. The global distribution and climate resilience of marine heterotrophic prokaryotes. Nature Communications 15: 6943. 10.1038/s41467-024-50635-z PMC11322184 39138161

[bib53] Huisman J, Codd GA, Paerl HW, Ibelings BW, Verspagen JMH, Visser PM. 2018. Cyanobacterial blooms. Nature Reviews Microbiology 16: 471–483. 29946124 10.1038/s41579-018-0040-1

[bib54] Hutchinson GE. 1961. The paradox of the plankton. American Naturalist 95: 137–145.

[bib56] [IPBES]Intergovernmental Science-Policy Platform on Biodiversity and Ecosystem Services . 2022. Summary for Policymakers of the Methodological Assessment of the Diverse Values and Valuation of Nature of the Intergovernmental Science-Policy Platform on Biodiversity and Ecosystem Services (IPBES). IPBES. www.ipbes.net/document-library-catalogue/summary-policymakers-methodological-assessment-regarding-diverse.

[bib57] Jönsson M, Hylander S, Ranåker L, Nilsson PA, Brönmark C. 2011. Foraging success of juvenile pike *Esox lucius* depends on visual conditions and prey pigmentation. Journal of Fish Biology 79: 290–297. 21722125 10.1111/j.1095-8649.2011.03004.x

[bib58] Jungck JR, Wagner R, van Loo D, Grossman B, Khiripet N, Khiripet J, Khantuwan W, Hagan M. 2019. Art Forms in nature: Radiolaria from Haeckel and Blaschka to 3D nanotomography, quantitative image analysis, evolution, and contemporary art. Theory in Biosciences 138: 159–187. 30868435 10.1007/s12064-019-00289-z

[bib59] Kiørboe T, Visser A, Andersen KH. 2018. A trait-based approach to ocean ecology. ICES Journal of Marine Science 75: 1849–1863.

[bib60] Kirby RR, Beaugrand G, Kleparski L, Goodall S, Lavender S. 2021. Citizens and scientists collect comparable oceanographic data: Measurements of ocean transparency from the Secchi Disk study and science programmes. Scientific Reports 11: 15499. 34326437 10.1038/s41598-021-95029-z PMC8322096

[bib61] Kotterba P, Moll D, Winkler H, Finke A, Polte P. 2024. A wolf in sheep's clothing: Planktivorous A tlantic herring preys on demersal fishes in coastal waters. Ecology 105: e4363. 38894614 10.1002/ecy.4363

[bib62] Krishnamurthy D, Li H, Benoit du Rey F, Cambournac P, Larson A, Prakash M. 2019. Scale-free Vertical tracking microscopy: Towards bridging scales in biological oceanography. 10.1038/s41592-020-0924-7 32807956

[bib63] Leone A, Lecci RM, Durante M, Meli F, Piraino S. 2015. The bright side of gelatinous blooms: Nutraceutical value and antioxidant properties of three Mediterranean jellyfish (Scyphozoa). Marine Drugs 13: 4654–4681. 26230703 10.3390/md13084654 PMC4556998

[bib64] Lombard F et al. 2019. Globally consistent quantitative observations of planktonic ecosystems. Frontiers in Marine Science 6.

[bib65] Macêdo RL, Franco ACS, Kozlowsky-Suzuki B, Mammola S, Dalu T, Rocha O. 2022. The global social-economic dimension of biological invasions by plankton: Grossly underestimated costs but a rising concern for water quality benefits? Water Research 222: 118918. 35932706 10.1016/j.watres.2022.118918

[bib66] Marten G. 2007. Larvicidal algae. Journal of the American Mosquito Control Association 23: 177–183. 10.2987/8756-971X(2007)23[177:LA]2.0.CO;2 17855939

[bib67] Martins Medeiros IP, Souza MM. 2023. Acid times in physiology: A systematic review of the effects of ocean acidification on calcifying invertebrates. Environmental Research 231: 116019. 37119846 10.1016/j.envres.2023.116019

[bib68] Mathijssen AJTM, Culver J, Bhamla MS, Prakash M. 2019. Collective intercellular communication through ultra-fast hydrodynamic trigger waves. Nature 571: 560–564. 31292551 10.1038/s41586-019-1387-9

[bib69] Michelutti N, Wolfe AP, Cooke CA, Hobbs WO, Vuille M, Smol JP. 2015. Climate change forces new ecological states in Tropical Andean Lakes. PLOS ONE 10: e0115338. 25647018 10.1371/journal.pone.0115338 PMC4315470

[bib70] Miller SM. 2010. Volvox, Chlamydomonas, Evolution of multicellularity | learn science at scitable. www.nature.com/scitable/topicpage/volvox-chlamydomonas-and-the-evolution-of-multicellularity-14433403

[bib71] Millette NC et al. 2023. Mixoplankton and mixotrophy: Future research priorities. Journal of Plankton Research 45: 576–596. 37483910 10.1093/plankt/fbad020 PMC10361813

[bib72] Mills MM, Ridame C, Davey M, La Roche J, Geider RJ. 2004. Iron and phosphorus co-limit nitrogen fixation in the eastern tropical North Atlantic. Nature 429: 292–294. 15152251 10.1038/nature02550

[bib73] Miloslavich P, et al . 2018. Essential ocean variables for global sustained observations of biodiversity and ecosystem changes. Global Change Biology 24: 2416–2433. 29623683 10.1111/gcb.14108

[bib74] Moreno AR, Martiny AC. 2018. Ecological stoichiometry of Ocean plankton. Annual Review of Marine Science 10: 43–69. 10.1146/annurev-marine-121916-063126 28853998

[bib76] Nature Conservation Bureau . 2011. Marine Biodiversity Conservation Strategy. Japanese Ministry of the Environment.

[bib77] Nicol S. 2006. Krill, currents, and sea ice: Euphausia superba and its changing environment. BioScience 56: 111–120.

[bib78] O'Connor S, Kenter J. 2019. Making intrinsic values work: Integrating intrinsic values of the more-than-human world through the Life Framework of Values. Sustainability Science 14: 1247–1265.

[bib79] Ogutu-Ohwayo R, Natugonza V, Musinguzi L, Olokotum M, Naigaga S. 2016. Implications of climate variability and change for African lake ecosystems, fisheries productivity, and livelihoods. Journal of Great Lakes Research 42: 498–510.

[bib80] O'Neill J. 1992. The varieties of intrinsic value. Monist 75: 119–137.

[bib81] Opdal AF, Lindemann C, Aksnes DL. 2019. Centennial decline in North Sea water clarity causes strong delay in phytoplankton bloom timing. Global Change Biology 25: 3946–3953. 31442348 10.1111/gcb.14810

[bib82] Pascual U et al. 2023. Diverse values of nature for sustainability. Nature 620: 813–823. 37558877 10.1038/s41586-023-06406-9 PMC10447232

[bib83] Perhar G, Arhonditsis GB, Brett MT. 2012. Modelling the role of highly unsaturated fatty acids in planktonic food web processes: A mechanistic approach. Environmental Reviews 20: 155–172.

[bib84] Picheral M et al. 2022. The Underwater Vision Profiler 6: An imaging sensor of particle size spectra and plankton, for autonomous and cabled platforms. Limnology and Oceanography: Methods 20: 115–129. 35909413 10.1002/lom3.10475 PMC9304221

[bib85] Pierella Karlusich JJ, Lombard F, Irisson J-O, Bowler C, Foster RA. 2022. Coupling imaging and omics in plankton surveys: State-of-the-art, challenges, and future directions. Frontiers in Marine Science 9: 878803.

[bib86] Platt T, Denman K. 1977. Organisation in the pelagic ecosystem. Helgoländer Wissenschaftliche Meeresuntersuchungen 30: 575–581.

[bib87] Pohl G, Nachtigall W. 2015. Biomimetics for Architecture and Design: Nature–Analogies–Technology. Springer International.

[bib88] Poloczanska ES et al. 2013. Global imprint of climate change on marine life. Nature Climate Change 3: 919–925.

[bib89] Ratnarajah L et al. 2023. Monitoring and modelling marine zooplankton in a changing climate. Nature Communications 14: 564. 10.1038/s41467-023-36241-5 PMC9895051 36732509

[bib90] Ravera O. 2001. Monitoring of the aquatic environment by species accumulator of pollutants: A review. Journal of Limnology 60: 63.

[bib91] Régnier P et al. 2015. Astaxanthin from *Haematococcus pluvialis* prevents oxidative stress on Human endothelial cells without toxicity. Marine Drugs 13: 2857–2874. 25962124 10.3390/md13052857 PMC4446609

[bib92] Riccio G, Lauritano C. 2019. Microalgae with immunomodulatory activities. Marine Drugs 18: 2. 31861368 10.3390/md18010002 PMC7024220

[bib93] Richardson AJ, Bakun A, Hays GC, Gibbons MJ. 2009. The jellyfish joyride: Causes, consequences, and management responses to a more gelatinous future. Trends in Ecology and Evolution 24: 312–322. 19324452 10.1016/j.tree.2009.01.010

[bib94] Rigby S, Milsom CV. 2000. Origins, evolution, and diversification of zooplankton. Annual Review of Ecology and Systematics 31: 293–313.

[bib96] Rojo C, Álvarez-Cobelas M, Benavent-Corai J, Barón-Rodríguez MM, Rodrigo MA. 2012. Trade-offs in plankton species richness arising from drought: Insights from long-term data of a National Park wetland (central Spain). Biodiversity and Conservation 21: 2453–2476.

[bib97] Ruggiero MA, Gordon DP, Orrell TM, Bailly N, Bourgoin T, Brusca RC, Cavalier-Smith T, Guiry MD, Kirk PM. 2015. A higher level classification of all living organisms. PLOS ONE 10: e0119248. 25923521 10.1371/journal.pone.0119248 PMC4418965

[bib98] Saini E, Rohilla P. 2020. Forensic diatomological mapping: A data base for diatom profiling to solve drowning cases. International Journal of Current Research and Review 12: 23–31.

[bib99] Sardet C. 2015. Plankton: Wonders of the Drifting World. University of Chicago Press.

[bib100] Scharlemann JPW et al. 2020. Towards understanding interactions between Sustainable Development Goals: The role of environment–human linkages. Sustainability Science 15: 1573–1584.

[bib101] Sharma N, Simon DP, Diaz-Garza AM, Fantino E, Messaabi A, Meddeb-Mouelhi F, Germain H, Desgagné-Penix I. 2021. Diatoms biotechnology: Various industrial applications for a greener tomorrow. Frontiers in Marine Science 8: 636613.

[bib102] Shimoda Y, Azim ME, Perhar G, Ramin M, Kenney MA, Sadraddini S, Gudimov A, Arhonditsis GB. 2011. Our current understanding of lake ecosystem response to climate change: What have we really learned from the north temperate deep lakes? Journal of Great Lakes Research 37: 173–193.

[bib103] Siciliano A, Gesuele R, Pagano G, Guida M. 2015. How *Daphnia* (Cladocera) assays may be used as bioindicators of health effects? Journal of Biodiversity and Endangered Species S1: 005.

[bib104] Siegel DA, DeVries T, Cetinić I, Bisson KM. 2023. Quantifying the ocean's biological pump and its carbon cycle impacts on global scales. Annual Review of Marine Science 15: 329–356. 10.1146/annurev-marine-040722-115226 36070554

[bib106] Skogen MD et al. 2021. Disclosing the truth: Are models better than observations? Marine Ecology Progress Series 680: 7–13.

[bib105] Skogen M et al. 2024. Bridging the gap: Integrating models and observations for better ecosystem understanding. Marine Ecology Progress Series 739: 257–268.

[bib107] Spanbauer TL, Briseño-Avena C, Pitz KJ, Suter E. 2020. Salty sensors, fresh ideas: The use of molecular and imaging sensors in understanding plankton dynamics across marine and freshwater ecosystems. Limnology and Oceanography Letters 5: 169–184.

[bib108] Steinberg DK, Landry MR. 2017. Zooplankton and the Ocean carbon cycle. Annual Review of Marine Science 9: 413–444. 10.1146/annurev-marine-010814-015924 27814033

[bib109] Suthers IM, Rissik D, Richardson A. 2019. Plankton: A Guide to Their Ecology and Monitoring for Water Quality, 2nd ed. CSIRO.

[bib110] Takenaka Y, Yamaguchi A, Shigeri Y. 2017. A light in the dark: Ecology, evolution and molecular basis of copepod bioluminescence. Journal of Plankton Research 39: 369–378.

[bib111] Tanioka T, Garcia CA, Larkin AA, Garcia NS, Fagan AJ, Martiny AC. 2022. Global patterns and predictors of C:N:P in marine ecosystems. Communications Earth and Environment 3: 1–9. 10.1038/s43247-022-00603-6 PMC9640808 36407846

[bib112] Tilman D. 1982. Resource Competition and Community Structure. Princeton University Press. 7162524

[bib113] Timsit Y, Lescot M, Valiadi M, Not F. 2021. Bioluminescence and photoreception in unicellular organisms: Light-signalling in a bio-communication perspective. International Journal of Molecular Sciences 22: 11311. 34768741 10.3390/ijms222111311 PMC8582858

[bib114] Toro-Farmer G, Muller-Karger FE, Vega-Rodríguez M, Melo N, Yates K, Cerdeira-Estrada S, Herwitz SR. 2016. Characterization of available light for seagrass and patch reef productivity in Sugarloaf Key, Lower Florida Keys. Remote Sensing 8: 86.

[bib115] Trubovitz S, Lazarus D, Renaudie J, Noble PJ. 2020. Marine plankton show threshold extinction response to Neogene climate change. Nature Communications 11: 5069. 10.1038/s41467-020-18879-7 PMC7582175 33093493

[bib116] Turner JT. 2015. Zooplankton fecal pellets, marine snow, phytodetritus and the ocean's biological pump. Progress in Oceanography 130: 205–248.

[bib117] Tyrrell T. 2008. Calcium carbonate cycling in future oceans and its influence on future climates. Journal of Plankton Research 30: 141–156.

[bib118] Varanasi U, Trainer VL, Schumacker EJ. 2021. Taking the long view for oceans and Human health connection through community driven science. International Journal of Environmental Research and Public Health 18: 2662. 33800838 10.3390/ijerph18052662 PMC7967353

[bib119] Vaughn D, Allen JD. 2010. The Peril of the Plankton. Integrative and Comparative Biology 50: 552–570. 21558224 10.1093/icb/icq037

[bib120] Wilkinson MD et al. 2016. The FAIR Guiding Principles for scientific data management and stewardship. Scientific Data 3: 160018. 26978244 10.1038/sdata.2016.18 PMC4792175

[bib121] Winder M, Sommer U. 2012. Phytoplankton response to a changing climate. Hydrobiologia 698: 5–16.

[bib122] Wolf R, Heuschele J. 2018. Water browning influences the behavioral effects of ultraviolet radiation on zooplankton. Frontiers in Ecology and Evolution 6: 26.

[bib123] Woolway RI, Kraemer BM, Lenters JD, Merchant CJ, O'Reilly CM, Sharma S. 2020. Global lake responses to climate change. Nature Reviews Earth and Environment 1: 388–403.

[bib124] Zhang C et al. 2022. Eco-engineering approaches for ocean negative carbon emission. Science Bulletin 67: 2564–2573. 36604035 10.1016/j.scib.2022.11.016

[bib125] Zohary T, Flaim G, Sommer U. 2021. Temperature and the size of freshwater phytoplankton. Hydrobiologia 848: 143–155.

