# Supplementary material for: The immeasurable value of plankton to humanity
Source: Bioscience. 2025 Jun 24;75(9):706–21. doi: 10.1093/biosci/biaf049 (PMC12412299; doi:10.1093/biosci/biaf049)
Supplement: biaf049_Supplemental_File [file biaf049_supplemental_file.docx]

**The immeasurable value of plankton to humanity.**

Maria Grigoratou, Susanne Menden-Deuer, Abigail McQuatters-Gollop, George Arhonditsis, Luis Felipe Artigas, Sakina-Dorothée Ayata, Dalida Bedikoğlu, Beatrix Beisner, Bingzhang Chen, Claire Davies, Lillian Diarra, Owoyemi W. Elegbeleye, Jason D. Everett Tatiane Garcia, Wendy C. Gentleman, Rodrigo Gonçalves, Tamar Guy-Haim, Svenja Halfter, Jana Hinners, Richard Horaeb, Jenny Huggett, Catherine L. Johnson L., Maria T. Kavanaugh, Ana Lara-Lopez, Christian Lindemann,Celeste López-Abbate, Monique Messié, Klas Ove Möller, Enrique Montes, Frank Muller-Karger, Aimee Neeley, Yusuf Olaleye, Artur Palacz, Alex J. Poulton, A. E. Friederike Prowe, Lavenia Ratnarajah, Luzmila Rodríguez, Clara Natalia Rodríguez-Flórez, Aurea Rodriquez-Santiago, Cecile S. Rousseaux, Juan Saad Francisco, Ioulia Santi, Alice Soccodato, Rowena Stern, Selina Våge, Ioanna Varkitzi, Anthony Richardson

The Supplementary Materials include three Tables.

The tables offer plankton-related examples of frugal science, citizen science, outreach actions and literacy (**Table SM1**), examples of how plankton has influenced and inspired various creative fields (**Table SM2**), and examples of plankton variables used in policy frameworks, laws, regulations, commercial catch agreements, and monitoring and assessment programmes directly linked to national laws or management actions across Africa, the Americas, Australia, Europe, and Japan (**Table SM3**).

**Table SM1**: **Examples of frugal science, citizen science, outreach actions and literacy all focused on plankton. We used the following definitions for the categorization:**

|  | **Frugal science**: An approach to scientific research that uses low-cost high-quality, simple tools and methods to study plankton and democratize science access. |
| --- | --- |
|  | **Citizen science**: A practice where non-professional scientists, often volunteers, contribute to plankton research by collecting data, observing, and analysing plankton samples to support scientific studies and monitoring programs. |
|  | **Outreach Activities**: Activities aimed at educating and engaging the public about plankton, such as workshops, presentations, or community events, to raise awareness and interest in marine science. |
|  | **Plankton Literacy**: The understanding and knowledge of plankton, including their types, roles in ecosystems, and importance, enabling individuals to appreciate their ecological significance and impact. |

| **Category**  **(type)** | **Title** | **Aquatic ecosystem** | **Summary** |
| --- | --- | --- | --- |
| **Frugal science (microscope)** | PlanktoScope | Freshwater & Marine | Planktoscope is an innovative, open-source microscope that was created to make the study of plankton more accessible and affordable. It allows users to automatically image and analyse plankton samples in water, providing high-quality pictures and data. Developed by a community of scientists and engineers, the Planktoscope is designed to be portable and user-friendly, enabling not just researchers but also students, citizen scientists, and environmental organizations to observe and monitor plankton. |
| **Frugal science (microscope)** | Foldscope | Freshwater & Marine | Foldscope is a low-cost, foldable paper microscope designed for portability and easy use. It was created to make science accessible to everyone, providing up to 140x magnification which makes it ideal for exploring tiny organisms like plankton. Foldscope offers an affordable way for researchers, students, and citizen scientists to observe and study plankton in the field. |
| **Citizen science** | Lakes of Missouri Volunteer Program (USA) | Freshwater | The Lakes of Missouri Volunteer Program engages citizens in collecting and processing water samples, ensuring research-quality data on Missouri's lake water quality through analysis at the University of Missouri. This citizen science initiative fosters collaboration and public involvement in monitoring and preserving Missouri's aquatic ecosystems. |
| **Citizen science** | Lake Observer | Freshwater | Lake Observer is a free app, created by the Global Ecological Observatory Network, aimed at enabling users to gather and share water quality data for their chosen water bodies. The app facilitates the collection of various observations such as Secchi-disc depth, ice cover, air temperature, precipitation, wind speeds, and details on plant species and algae. Data can be recorded year-round and is submitted to citsci.org for citizen science purposes, with users receiving email notifications about their submission status. |
| **Citizen science** | Secchi Disk study | Freshwater & Marine | The Secchi Disk Foundation's global citizen science project is the largest phytoplankton citizen science program in the world. It engages participants in collecting ocean transparency data using homemade Secchi Disks and a smartphone app, helping assess climate-induced changes in phytoplankton. |
| **Citizen science** | Phytoplankton Monitoring Network  (USA) | Freshwater & Marine | NOAA's Phytoplankton Monitoring Network (PMN) enlists over 600 volunteers across 36 U.S. states and territories to monitor phytoplankton and harmful algal blooms. It provides vital data for NOAA forecasts and management efforts. |
| **Citizen science** | OBIS -eDNA expeditions | Marine | The eDNA expeditions initiative (2022-2023) will collect and sequence DNA from 500 water samples across 25 marine World Heritage sites, with all data made publicly available through OBIS to enhance ocean protection and standardize eDNA practices. This global project aims to standardize eDNA collection and processing while ensuring open access to data, marking a crucial step in advancing ocean protection amid climate change. |
| **Citizen science** | FjordPhyto programme  (Antarctica) | Marine | FjordPhyto, initiated in 2015, examines how glacier melting affects Antarctic phytoplankton by collecting and analysing data on seawater conditions and phytoplankton diversity, and is creating a field guide for species identification. |
| **Citizen science** | El Veril (Argentina) | Marine | The "El Veril" project is a collaboration between INIDEP and the CASE diving club to monitor ocean acidification near Mar del Plata, Argentina. CASE divers collect and analyse seawater samples for the analysis of various parameters including chlorophyll-a, phytoplankton, and bacterioplankton abundance, and document environmental conditions during their recreational dives at a coastal site known for its marine biodiversity. This effort aims to track potential changes in the marine environment due to acidification. |
| **Citizen science** | Jelly Spotter by GoJelly  (Baltic Sea) | Marine | The app features photos of common Baltic Sea jellyfish for easy species identification. When uncertain about the species, users can upload pictures in a dedicated category for the experts to identify them. |
| **Citizen science** | Jellyfish and sea turtle  (Canada) | Marine | A citizen science program in Atlantic Canada (2007 – 2010) tracked jellyfish and leatherback sea turtles by analysing over 23,600 stranded jellyfish sightings, revealing how jellyfish distributions and environmental factors influence leatherback turtle movements and habitat use. |
| **Citizen science** | Pacific Salmon Foundation Citizen Science  (Canada) | Marine | Launched in 2015, the Pacific Salmon Foundation (PSF) Citizen Science program engages volunteers using their own fishing vessels to conduct oceanographic surveys in the Strait of Georgia, collecting data on temperature, salinity, plankton productivity, and water quality. This data is transmitted via a custom app, processed, and made publicly available through the PSF-UBC Strait of Georgia Data Centre, supporting scientific research and public outreach. |
| **Citizen science** | Hong Kong Jellyfish Project  (China) | Marine | The Hong Kong Jellyfish Project, initiated in 2021, compiled 1,020 citizen-reported jellyfish sightings from 2021 to 2023, creating a publicly available dataset that enhances understanding of jellyfish distribution in local waters. |
| **Citizen science** | Objective Plankton (Objectif Plancton) Programme  (France) | Marine | Objective Plankton, coordinated by Océanopolis and the Concarneau Marine Station, involves volunteers and local groups in sampling plankton across Brest, Lorient, and Concarneau Bay three times a year. This citizen science initiative aims to study seasonal variations and distributions of plankton and fish larvae to better understand coastal and ocean ecosystems. |
| **Citizen science** | Real-Time Monitoring of Lake Water Quality  (India) | Marine | A citizen science network in Vembanad Lake, involving residents and students, by using mini Secchi disks and the "TurbAqua" app to monitor water quality by measuring colour and clarity. This low-cost initiative helps track water conditions, supports sustainable monitoring, and aligns with Sustainable Development Goal 6 for clean water. |
| **Citizen science (sensors and cameras)** | SOOP Innovation Platform | Marine | Equip citizen yachts, sailboats, and cruise ships with sensors and cameras for microplastic and plankton observations. Develop low-cost sensors and connect sensor manufacturers with citizen scientists. |
| **Citizen science** | Infomedusa app  (Spain) | Marine | Infomedusa is an app that provides real-time updates on jellyfish presence and beach conditions in Spain, helping users choose beaches. |
| **Citizen science** | MEDJELLYRISK project  Tunisia | Marine | Launched in 2013, the MEDJELLYRISK Project enhances citizen science by distributing identification guides and conducting training for stakeholders and the public, while also providing jellyfish information and stinging treatment on a dedicated Facebook page. |
| **Citizen science** | Monitoring Anthropogenic Particles and Plankton project  (UK) | Marine | The MAPPS project engages citizen scientists to monitor microplastics and plankton using a low-cost PlanktoScope microscope, aiming to create a classifier for identifying synthetic particles and assessing microplastic contamination in UK rivers and coasts. |
| **Citizen science** | Gallery of Maine Jellyfish Sightings  (USA) | Marine | The programme uses citizen scientist reports of jellyfish to track jellyfish along the coast of Maine. Citizens can email jellyfish sightings including date, time, and location, as well as any descriptive information such as size or type. |
| **Citizen science** | PlanktonPlanet | Marine | Plankton Planet is a citizen science project involving sailors collecting and imaging plankton worldwide. Participants receive training, build their own Planktoscope—a low-cost microscope to capture microplankton images—and use a sampling toolkit to gather planktonic DNA for sequencing. The collected images are uploaded to EcoTaxa for classification, contributing to global plankton research. |
| **Outreach activity (video game)** | Plankton Game by PlanktonID | Marine | Plankton Game is part of the Plankton ID platform developed by GEOMAR. This interactive game is designed to help users learn more about plankton species and improve their identification skills. Players are tasked with identifying various plankton based on images and information provided. |
| **Outreach activity (video game)** | Bionix: Spore Evolution Sim 3D | Freshwater & Marine | The game simulates the life of real underwater organisms such as diatoms, ciliates, bacteria, bacilli, spirochetes, and algae. It allows the user to create a custom creature, consume cells and microbes, gather DNA, and evolve by upgrading stats and mutations while battling enemies. |
| **Outreach activity** | Biobus  (USA) | Freshwater & Marine | BioBus helps K-12 and college students discover, explore, and pursue science. Students use microscopes to examine and observe micro- and macroscopic organisms, including plankton, and learn about their ecology. |
| **Outreach activity**  **(Virtual Reality application)** | PlanktonQuest | Marine | PlanktoQuest is a virtual reality animation to explore and manipulate marine plankton with hands. |
| **Outreach activity (Hands-on activity)** | The Great Plankton Race  (USA) | Marine | The objective of the action is for students to use a variety of materials to construct various models of plankton to understand neutral buoyancy for children ages 4-14. |
| **Outreach activity** | Plankterception: Dancing with Chaos  (USA) | Marine | *Plankterception* is an interactive art installation and STEAM lesson plan designed to raise ocean awareness during the UN's Decade of Ocean Science. It engages elementary students with plankton and turbulence through visual and kinesthetic learning, enhancing public engagement with science, and was introduced to San Diego teachers for World Oceans Day. |
| **Plankton literacy and outreach** | Jellyfish education and outreach  (South Africa) | Marine | Educational website for information on jellyfish, which supports the permanent live jellyfish exhibit at the Two Oceans Aquarium in Cape Town. |
| **Plankton literacy and outreach** | The Plankton Manifesto - A call for Plankton-Based Solutions to address The Triple Planetary Crisis (biodiversity, climate & pollution) | Marine | The *Plankton Manifesto* calls for global recognition and action to protect plankton, essential yet threatened organisms. It advocates for the adoption of "Plankton-Based Solutions" to benefit humanity, leveraging technological advances to deepen our understanding and improve the stewardship of plankton. Led by the UN Global Compact and 30 experts, it seeks endorsements at key environmental conferences, including COP29, COP16, and the 2025 UN Ocean Conference. |
| **Plankton literacy and outreach**  **(exhibition)** | JellyWorld: Jellyfish education and outreach  (South Africa) | Marine | Museum exhibition and multimedia display focused on the interaction between humans and jellyfish, showcasing cutting-edge research by local jellyfish scientists and partners. At Iziko Museum, Cape Town. |
| **Plankton literacy (blog)** | PlanktonOcean | Marine | PlanktonOcean is an online resource dedicated to the study and appreciation of plankton. It offers a variety of content related to planktonic organisms, including educational materials, research updates, and multimedia resources. The platform aims to provide information on the ecological roles of plankton, their importance in marine ecosystems, and recent scientific findings, making it a valuable tool for both researchers and the general public interested in marine biology. |
| **Plankton literacy (book)** | Plankton: A Worldwide Guide by Tom Jackson and Jennifer Parker | Marine | A richly illustrated guide to the marvellously diverse plankton of the world and their fundamental role in planetary food webs. |
| **Plankton literacy (book)** | Ocean Drifters: A Secret World Beneath the Waves by Richard R. Kirby | Marine | Richard Kirby's high-magnification photographs and text reveal the beauty and diversity of plankton, highlighting their crucial role in the marine food chain, oxygen production, and the carbon cycle while examining the impact of global change and rising sea temperatures on plankton communities. |
| **Plankton literacy (book)** | Planktonia: The Nightly Migration of the Ocean's Smallest Creatures by Erich Hoyt | Marine | In Planktonia, Erich Hoyt invites readers to explore the fascinating vertical migration of plankton and their predators, who ascend to the ocean's surface at night to feed before returning to the depths by sunrise. |
| **Plankton literacy (book)** | Planktonium: An Unseen World by Jan van Ijken | Marine | Planktonium is a photo project and short film by Jan van IJken that unveils the hidden world of microscopic plankton, showcasing their stunning beauty and vital ecological roles, while highlighting their vulnerability to climate change and ocean acidification. |
| **Plankton literacy (book)** | Plankton (Earth's Smallest Superheroes) by Ruth Owen | Marine | This book uses photos, 3D artwork, and SEM imagery to present the crucial role of plankton in marine ecosystems, highlighting their diverse species, their importance in marine life cycles, and their role in oxygen production and carbon dioxide absorption. |
| **Plankton literacy (book)** | Plankton: Wonders of the Drifting World by Christian Sardet | Marine | This book reveals the vibrant and intricate world of plankton through hundreds of close-up photographs. It also explains their biological significance, diverse species, and crucial role in the ocean's ecosystem. |
| **Plankton literacy (book)** | A Guide to Their Ecology and Monitoring for Water Quality Second Edition edited by Iain M Suthers, David Rissik, Anthony J Richardson | Marine | The second edition of *Plankton* offers a comprehensive update on the biology, ecology, and identification of plankton, highlighting their crucial role in monitoring water quality and supporting aquatic ecosystems. The book is an invaluable reference for teachers and students, environmental managers, ecologists, estuary and catchment management committees, and coastal engineers. |
| **Plankton literacy (children's book)** | Gigantes microscópicos by Paula de Tezanos Pinto | Freshwater & Marine | The book examines the ecology of cyanobacteria and their blooms, explaining how scientists utilize satellite imagery and water samples to study them in both freshwater and marine ecosystems. |
| **Plankton literacy (children's book)** | Sea Soup: Phytoplankton by Mary M. Cerullo | Marine | The book explores the fascinating world of phytoplankton, highlighting their vital role in ocean ecosystems with stunning colour photography that captures the diversity of these microscopic creatures. It has received the Notable Books for Children, 1999 - Smithsonian Outstanding 1999 Books awards. |
| **Plankton literacy (children's book)** | Sea Soup: Zooplankton by Mary M. Cerullo | Marine | The book reveals the fascinating world of zooplankton, showcasing their diversity, from glowing species to deadly jellyfish, and their crucial role in the ocean food web. It received the Smithsonian Notable Books for Children award, the Outstanding Science Trade Books for Children award by the Children’s Book Council/NSTA and the Honor Book award by the Society of School Librarians International. |
| **Plankton literacy (children's book)** | The adventures of Pepo the copepod by Albert Calbet | Marine | In this book, Pepo offers an educational introduction to copepods and their role in the sea. It features two levels of reading: one for children aged 6 and up, and another for adults seeking more detailed information about marine plankton. The book aims to raise awareness about the fragility and importance of plankton and the need to protect the ocean. |
| **Plankton literacy (children's interactive online story)** | Otto the copepod by Jan Heuschele and the Centre of Ocean Life | Marine | Otto the Copepod is an interactive story where Otto learns about other plankton and marine species and sinking particles while migrating from the surface to deeper waters. |
| **Plankton literacy (coloring book)** | Artscipaedia Plankton by Judy D. Lemus | Marine | The Plankton Artscipaedia is a colouring book designed to combine art and science, focusing on the diverse world of plankton. It allows users to colour detailed illustrations of various plankton species with an aim to raise awareness about the ecological importance of plankton in a fun and engaging way. |
| **Plankton literacy (video)** | Journey to the microcosmos | Freshwater & Marine | Journey to the microcosmos provides educational videos about plankton species and their ecology. |
| **Plankton literacy (video)** | The Secret Life of Plankton | Marine | The Secret Life of Plankton TED-Ed video presents the dynamic world of plankton and illustrates the complexity of microscopic life through the lifecycle of a fish, from its hatching as an egg to reaching adulthood. |
| **Plankton literacy (video)** | How Life Begins in the Deep Ocean | Marine | This TED-Ed video explores various reproductive strategies of plankton, featuring a sea urchin larva as the central focus. |
| **Plankton literacy (video)** | Ocean Drifters | Marine | In "Ocean Drifters," David Attenborough sheds light on the vital role plankton plays in marine ecosystems and their surprising connection to the formation of oil and gas resources. |
| **Plankton literacy (video)** | Plankton Chronicles | Marine | The Plankton Chronicles project is a short documentary series combining art and science, revealing the beauty and diversity of plankton. |
| **Plankton literacy (video)** | Gravity Machine Gallery | Marine | The Gravity Machine Gallery provides videos that allow us to discover the behaviours of marine invertebrate larvae and single-celled protists, captured at a multi-scale resolution via the Gravity Machine, a Scale-free Vertical Tracking Microscopy. |

**Table SM2: Examples of how plankton has influenced and inspired various creative fields, including visual art, children's literature, interior design, fashion, and stamps. The “children’s literature” section in Table SM2 lists books with fictional characters inspired by plankton shapes. See Table SM1 for book examples designed to educate readers about plankton species and their ecology.**

| **Type of creation** | **Title/Creator** | **Country of origin** |
| --- | --- | --- |
| **Pop culture** | SpongeBob Square Pants TV show and movie by Stephen Hillenburg | USA |
| **Children's Literature** | The Story of Plankton by Jackie Robb and Berny Stringle | UK |
|  | Plankton Is Pushy by Jonathan Fenske | USA |
|  | The Crabby book series by Jonathan Fenske | USA |
| **Music** | Silent Plankton by Akira Sakata | Japan |
|  | Plankton by Ryuichi Sakamoto | Japan |
| **Visual Arts** | Bioluminescence-inspired events by Glowee | France |
|  | Boxes and sculptures by Louise Hibbert and Sarah Parker-Eaton | UK |
|  | Historical chandelier by Timothy Horn | Australia |
|  | From Plankton to human bodies: the dance of interacting types by Silvia De Monte, Adrienne Nowak, Marcela Moura, Florestan Labourdette, Pierre Carré | France |
|  | Moving Creates Vortices by TeamLab | Japan |
|  | Drawings by Ernst Haeckel | Germany |
|  | Glass art by William Geffroy | France |
|  | Glass sculpture by Léopold & Rudolf Blaschka | Germany |
|  | Graphics and digital art by Ellen Karin Mæhlum | Norway |
|  | Plankton Sculptures by Daisy Braun | USA |
|  | The medusa chandelier by Constant Roux and Maison Baguès | France |
|  | The Radiolaria chandelier by Maison Baguès | France |
|  | Painting by Cynthia Beth Rubin | USA |
|  | Paintings and textiles by Holly Sumner | USA |
|  | Photography by Klaus Kemp | UK |
|  | Photography by Wim van Egmond | Netherlands |
|  | Photography by Christian Sardet | France |
|  | Photography and digital art by Shiro Takatani | Japan |
|  | Plankton Ballet Ensemble video by Ai Nihongi and J. Rudi Strickler | UK |
|  | Sculptures by Katja Loher | Switzerland/USA |
|  | Sculptures by Shiro Studio | Italy |
|  | Sculptures by Nicoles Anona | Poland |
| **Interior design** | Diatom chair by Ross Lovegrove | UK (1958) |
|  | Tomopteris chandelier by Charles Prograce | USA |
|  | Plankton Lamp by Marta Cherednik | Australia |
|  | Diatom Lights by David Trubridge | Germany |
|  | Radiolaria Table by Il Hoon Roh | South Korea |
| **High Fashion** | Sensory Seas collection by Iris van Herpen | Netherlands |
| **Jewellery** | Artists draw inspiration from the unique shapes of plankton to craft intricate jewellery designs. Additionally, opal, a popular gemstone, forms from silica-based organisms like diatoms, linking plankton in the field of jewellery. | Various countries |
| **Textiles** | Companies and small textile businesses are creating textiles featuring plankton designs. | Various countries |
| **Stamps** | Countries worldwide have recognized the importance of plankton by featuring them on postage stamps, acknowledging their vital role in marine ecosystems and fostering global awareness. Cnidaria are the most common taxa used, followed by krill (Euphausia superba). Other taxa: Protozoa (Noctiluca), Radiolaria, Foraminifera, Ctenophora, Annelida, Copepoda, Decapoda (crab larvae, phyllosoma), Stomatopoda, Mysida, Amphipoda, Mollusca (Gastropoda, Cephalopoda), Echinodermata (larvae), Thaliacea, Chordata (larval fish). | Albania, Antigua & Barbuda, Ascension Island, Australia, Australian Antarctic Territory, Azerbaijan, Belgium, British Antarctic Territory, Bulgaria, Canada, China, Cuba, Falkland Islands, Faroe Islands, France (incl. Saint Pierre & Miquelon), French Southern and Antarctic Lands, Gambia, Greece, Greenland, Grenada, Iceland, Japan, Malaysia, Malta, Marshall Islands, Monaco, Mozambique, Namibia, Netherlands, Palau, Poland, Portugal, Ross Dependency, Saint Vincent and the Grenadines, San Marino, Senegal, Solomon Islands, Somalia, South Africa, South Georgia and the South Sandwich Islands, Suriname, Tanzania, Togo, Tonga, Tuvalu, Uruguay, UK, USA |

**Table SM3**: **Examples of plankton variables used in policy frameworks, laws, regulations, commercial catch agreements, and monitoring and assessment programmes directly linked to national laws or management actions across Africa, the Americas, Australia, Europe, and Japan. We note that in some documents referred to in the Table, plankton variables are used as indicators of specific conditions or statuses and may often be referred to as indicators rather than variables. For consistency throughout our article, in this Table we refer to them as plankton variables, not indicators.**

| 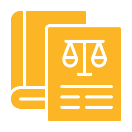 | Policy frameworks, laws, legislations, and agreements are a set of rules and strategies for managing and protecting marine environments and resources agreed on a national or international level. |
| --- | --- |
| 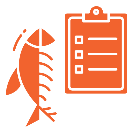 | Commercial catch agreements for plankton are regulations and treaties that govern the harvesting,  management, and conservation of fishery resources. |
| 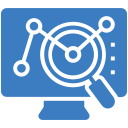 | Monitoring, regulatory, and assessment programmes are programmes directly linked with national laws or/and policy actions (e.g., direct management measures). |

|  | **Title** | **Country/**  **Countries/**  **Regions** | **Aquatic habitat** | **Plankton variables** | **Short summary and reference** |
| --- | --- | --- | --- | --- | --- |
| 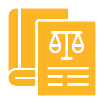 | Murray-Darling Basin Plan | Australia | Freshwater | phytoplankton and blue-green algae data | The Murray-Darling Basin Plan is a strategic framework developed by the Australian Government to ensure the sustainable management of water resources within the Murray-Darling Basin. Phytoplankton samples and satellite data are used to monitor phytoplankton concentrations and for blue-green algae alerts produced by the States.  *Reference: Monitoring Statement January 2021, Murray‒Darling Basin Authority Canberra, 2021,* [*https://www.mdba.gov.au/publications-and-data/publications/monitoring-statement*](https://www.mdba.gov.au/publications-and-data/publications/monitoring-statement) |
| 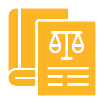 | Great Lakes Water Quality Agreement | Canada and USA | Freshwater | phytoplankton abundance, diversity, community composition, zooplankton abundance and community composition | The Great Lakes Water Quality Agreement is a prominent framework that provides a structured approach for Canada and the USA to collaborate on protecting the Great Lakes. The Agreement includes different facets of plankton ecology as a multitude of proxy variables to characterise impaired conditions, identify desirable quality standards, guide restoration efforts, and track progress toward their achievability.  *Reference: Government of Canada, Government of the United States. 2012. Great Lakes Water Quality Agreement. Ottawa (ON): Government of Canada; Washington (DC): Government of the United States,* [*https://www.epa.gov/glwqa*](https://www.epa.gov/glwqa) |
| 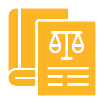 | South African Water Quality Guideline for Domestic Water Use | South Africa | Freshwater | chlorophyll *a*, phytoplankton community composition (categorised in the groups of blue-green algae, green algae, euglena, and diatoms), total coliform bacteria, faecal coliform bacteria (including *Vibrio cholerae*), *E. coli, Enterococci*, and bacteriophages. | These technical guidelines provide a foundation for sector-specific material development, enabling water users to understand water quality requirements and actively engage in management processes.  *Reference: Department of Water Affairs and Forestry. 1996. South African Water Quality Guidelines (2nd edition). Volume 1: Domestic Use. CSIR Environmental Services, Pretoria. 197pp.* [*https://www.iwa-network.org/filemanager-uploads/WQ_Compendium/Database/Selected_guidelines/041.pdf*](https://www.iwa-network.org/filemanager-uploads/WQ_Compendium/Database/Selected_guidelines/041.pdf) |
| 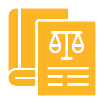 | South African Water Quality Guidelines for Aquatic Ecosystems | South Africa | Freshwater | Algae: chlorophyll *a*, phytoplankton community composition (categorised in the groups of blue-green algae, green algae, euglena, and diatoms) Variable organisms: heterotrophic bacteria, total coliforms (includes *Vibrio cholerae*), et al. | The South African Water Quality Guidelines for Aquatic Ecosystems serve as a key reference and decision-making tool for the Department of Water Affairs and Forestry in managing and protecting aquatic ecosystems. It specifies water quality requirements and criteria to protect freshwater ecosystems from toxic and non-toxic constituents, preserve ecosystem structure and function, manage nutrient-driven trophic changes, and permit site-specific adjustments without compromising protection levels. Plankton populations are mentioned as variables of water quality and ecosystem changes, and for the impacts of pollutants on these organisms.  *Reference: Department of Water Affairs and Forestry, 1996. South African Water Quality Guidelines. Volume 7: Aquatic Ecosystems,* [*https://www.dws.gov.za/iwqs/wq_guide/edited/Pol_saWQguideFRESH_vol7_Aquaticecosystems.pdf*](https://www.dws.gov.za/iwqs/wq_guide/edited/Pol_saWQguideFRESH_vol7_Aquaticecosystems.pdf) |
| 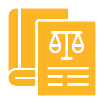 | Water Quality Improvement Plans | Australia | Freshwater and Marine | chlorophyll *a*, phytoplankton species, abundance, toxins, HABs | Plankton indicators are used for monitoring the water quality issues (e.g., eutrophication, toxins) and long-term water quality objectives (e.g., nutrient concentrations) for the targeted environmental values (e.g., ecosystem health, harvesting, cultural and spiritual).  *Reference: Department of Environment and Science, 2017. Water Quality Improvement Plan for the XYZ Catchment. Queensland Government,*  [*https://www.wa.gov.au/government/document-collections/water-quality-improvement-plans*](https://www.wa.gov.au/government/document-collections/water-quality-improvement-plans) |
| 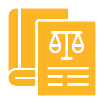 | Species at Risk Act | Canada | Freshwater and Marine | plankton are considered as an element of critical habitat under the Species at Risk Act, e.g., *Calanus spp*. as prey for endangered North Atlantic right whales. | Species at Risk management, protection of critical habitat for species at risk.  *Reference: Canada, 2002. Species at Risk Act (S.C. 2002, c. 29),* [*https://laws.justice.gc.ca/eng/acts/S-15.3/index.html*](https://laws.justice.gc.ca/eng/acts/S-15.3/index.html) |
| 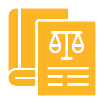 | EU Water Framework Directive | European Union (Austria, Belgium, Bulgaria, Croatia, Republic of Cyprus, Czech Republic, Denmark, Estonia, Finland, France, Germany, Greece, Hungary, Ireland, Italy, Latvia, Lithuania, Luxembourg, Malta, Netherlands, Poland, Portugal, Romania, Slovakia, Slovenia, Spain and Sweden) | Freshwater and Marine | Marine and coastal: Chlorophyll, phytoplankton blooms, diatom and dinoflagellate abundance. Quality Element Phytoplankton: composition, abundance, biomass | The WFD requires Member States to use their River Basin Management Plans (RBMPs) and Programmes of Measures (PoMs) to protect and, where necessary, restore water bodies in order to reach good status and to prevent deterioration. Good status means both good chemical and good ecological status. European directive requiring member states to protect and restore inland, transitional and coastal surface waters as well as groundwaters to reach good chemical and ecological status. Phytoplankton composition, abundance and biomass are monitored regularly in the coastal seas and transitional waters from rivers to the sea to detect high, good or moderate ecological status according to threshold values.  *Reference: European Parliament, & Council. (2000). Directive 2000/60/EC of the European Parliament and of the Council of 23 October 2000 establishing a framework for community action in the field of water policy. Official Journal of the European Communities, L 327, 1–73,* [*https://environment.ec.europa.eu/topics/water/water-framework-directive_en*](https://environment.ec.europa.eu/topics/water/water-framework-directive_en) |
| 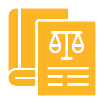 | International Convention for the Control and Management of Ships' Ballast Water and Sediments | International | Freshwater and Marine | toxicogenic *Vibrio cholerae* concentration in samples of zooplankton biomass, number of liable plankton organisms per cube meter | The plankton variables are used for tracking the concentration of toxicogenic *Vibrio cholerae* in samples of zooplankton biomass number of liable plankton organisms per cube meter  *Reference: International Maritime Organization, 2004. International Convention for the Control and Management of Ships’ Ballast Water and Sediments (BWM),* [*Regulation D-2 Ballast Water Performance Standard https://www.imo.org/en/About/Conventions/Pages/International-Convention-for-the-Control-and-Management-of-Ships%27-Ballast-Water-and-Sediments-(BWM).aspx*](Regulation%20D-2%20Ballast%20Water%20Performance%20Standard%20https://www.imo.org/en/About/Conventions/Pages/International-Convention-for-the-Control-and-Management-of-Ships%27-Ballast-Water-and-Sediments-(BWM).aspx) |
| 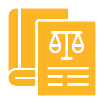 | Conservation of the Water Environment (Water Pollution / Present Status and Countermeasures) | Japan | Freshwater and Marine | phytoplankton blooms | The Conservation of the Water Environment has two goals: the protection of human health and the conservation of the living environment. Phytoplankton blooms are used as a factor for defining the environmental quality standards for nutrients (nitrogen, phosphorus).  *Reference: Japan Ministry of the Environment, 2020. Conservation of the water environment (water pollution / present status and countermeasures),* [*https://www.env.go.jp/en/water/wq/wemj/water.html#3-1*](https://www.env.go.jp/en/water/wq/wemj/water.html#3-1) |
| 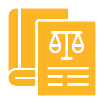 | UK Water Environment Regulations | UK | Freshwater and Marine | (for marine and coastal habitats only) Chlorophyll *a*, phytoplankton blooms, diatom and dinoflagellate abundance | The core aim of the Water Framework Directive is to protect the UK's water environments by preventing their deterioration and improving their quality. The framework includes plankton variables only for marine and coastal habitats.  *Reference: UK Government, 2017. The Water Environment (Water Framework Directive) (England and Wales) Regulations 2017,* [*https://www.legislation.gov.uk/uksi/2017/407/contents*](https://www.legislation.gov.uk/uksi/2017/407/contents) |
| 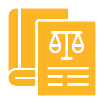 | United Nations Environmental Plan Mediterranean Action Plan (UNEP/MAP) | Albania, Algeria, Bosnia and Herzegovina, Croatia, Cyprus, Egypt, France, Greece, Israel, Italy, Lebanon, Libya, Malta, Monaco, Montenegro, Morocco, Slovenia, Spain, Syrian Arab Republic, Tunisia, Türkiye, and the European Union | Marine | phytoplankton (abundance, primary productivity), zooplankton (abundance, biomass, and biodiversity) | Plankton is monitored in the Mediterranean in the framework of UNEP/MAP (stemmed from the Barcelona Convention), which is compatible with the EU MSFD. These regulations follow the ecosystem approach (ECAP) with the aim to achieve and maintain Good Environmental Status (GES).  *Reference: UNEP/MAP, 1976. Convention for the Protection of the Marine Environment and Coastal Region of the Mediterranean (Barcelona Convention),* [*https://www.unep.org/unepmap/resources*](https://www.unep.org/unepmap/resources) |
| 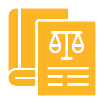 | Marine Strategy Framework Directive | European Union (Austria, Belgium, Bulgaria, Croatia, Republic of Cyprus, Czech Republic, Denmark, Estonia, Finland, France, Germany, Greece, Hungary, Ireland, Italy, Latvia, Lithuania, Luxembourg, Malta, Netherlands, Poland, Portugal, Romania, Slovakia, Slovenia, Spain and Sweden) | Marine | chlorophyll *a*, phytoplankton community composition, HABs, zooplankton biomass, zooplankton community structure and diversity, zooplankton size-structure, plankton phenology | The Marine Strategy Framework Directive assesses the environmental status of all European seas. The plankton variables are used under the descriptors D1C6 Biodiversity: Pelagic habitats, D4 Food webs, D5 Eutrophication. The development of variables includes thresholds in cooperation with OSPAR (<https://www.ospar.org/work-areas/cross-cutting-issues/qsr2023>) and HELCOM assessments (<https://helcom.fi/baltic-sea-trends/holistic-assessments/state-of-the-baltic-sea-2023/>).  *Reference: European Commission, 2008. Directive 2008/56/EC of the European Parliament and of the Council of 17 June 2008 establishing a framework for community action in the field of marine environmental policy (Marine Strategy Framework Directive). Official Journal of the European Union, L 164, 19–40,* [*https://eur-lex.europa.eu/eli/dir/2008/56/oj*](https://eur-lex.europa.eu/eli/dir/2008/56/oj) |
| 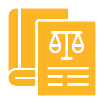 | OSPAR | Belgium, Denmark, Finland, France, Germany, Iceland, Ireland, Luxembourg, The Netherlands, Norway, Portugal, Spain, Sweden, Switzerland, United Kingdom, European Union | Marine | abundance, diversity, biomass, and community structure of phytoplankton and zooplankton | OSPAR is a regional agreement where member countries can collaborate on the protection and conservation of the North-East Atlantic marine environment. The agreement includes the development and implementation of policies, strategies, and actions to address various marine environmental issues, such as pollution, biodiversity loss, and climate change.  *Reference: OSPAR Commission. (2023). Quality Status Report, 2023,* [*https://oap.ospar.org/en/ospar-assessments/quality-status-reports/qsr-2023/*](https://oap.ospar.org/en/ospar-assessments/quality-status-reports/qsr-2023/) |
| 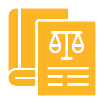 | Fisheries Act | Canada | Marine | chlorophyll *a*, phytoplankton community composition and abundance, zooplankton community composition, abundance, and biomass. | Zooplankton are included within the definition of Fish under the Fisheries Act (Section 2(1) b,c), and they may be considered in various decisions that address the productivity of fisheries and fish habitat (Section 34 Fish and Fish Habitat Protection and Pollution Prevention). Plankton variables may be included in fisheries stock assessments and in advice related to other industries that affect fisheries, fish, and fish habitat.  *Reference: Canada, 1996. Fisheries Act, RSC 1996, c 14,* [*https://laws.justice.gc.ca/eng/acts/F-14/*](https://laws.justice.gc.ca/eng/acts/F-14/) |
| 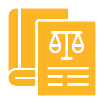 | Oceans Act | Canada | Marine | chlorophyll *a,* zooplankton community composition and abundance. | Section 35 Marine Protected Areas. Marine Protected Areas, designated for example for the protection of living marine resources or threatened/endangered species and their habitats, protection of unique habitats or areas of high biodiversity or productivity, or to protect areas to maintain ecological integrity. Plankton are considered in Marine Protected Area network planning, site designation, and monitoring plans under the Oceans Act.  *Reference: Canada, 1996. Oceans Act, RSC 1996, c 31,* [*https://laws-lois.justice.gc.ca/eng/acts/O-2.4/*](https://laws-lois.justice.gc.ca/eng/acts/O-2.4/) |
| 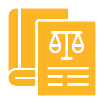 | Baltic Sea Action Plan (BSAP) | HELCOM (Denmark, Estonia, European Union, Finland, Germany, Latvia, Lithuania, Poland, Russia, Sweden) | Marine | chlorophyll *a*, cyanobacterial bloom index, diatom/dinoflagellate index, zooplankton mean size and total stock | HELCOM is a regional sea convention and policy framework, for contracting parties to collaborate and develop policies, strategies, and actions aimed at protecting the marine environment of the Baltic Sea. The plankton variables are being used for monitoring the status of pelagic habitats in regular assessment reports (e.g., HOLAS 3, 3rd HELCOM Holistic Assessment of the Baltic Sea 2016-2021). Many HELCOM variables are also used for national assessments of good environmental status of the European Seas within the scope of the Marine Strategy Framework Directive.  *Reference: HELCOM, 2021. Baltic Sea Action Plan, Reaching Good Environmental Status for the Baltic Sea,* [*https://helcom.fi/baltic-sea-action-plan/*](https://helcom.fi/baltic-sea-action-plan/) |
| 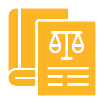 | Marine Biodiversity Conservation Strategy | Japan | Marine | phytoplankton and zooplankton | The Marine Biodiversity Conservation Strategy, led by the Ministry of the Environment, is a cross-sectoral policy aimed at protecting marine biodiversity, ensuring the sustainable use of ecological services, and enhancing the resilience of livelihoods through adaptive conservation management and governance. Phytoplankton and zooplankton are recognized as characteristic biological variables of various marine ecosystems of the Japanese marine zones (e.g., Kuroshio Current and Subtropical Zone and the Seto Inland Sea).  *Reference: Nature Conservation Bureau, Ministry of the Environment, Japan 2011. Marine Biodiversity Conservation Strategy, 58pp,* [*https://www.env.go.jp/content/900489567.pdf*](https://www.env.go.jp/content/900489567.pdf) |
| 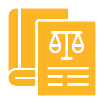 | South African Water Quality Guidelines for Coastal Marine Waters - Natural Environment and Mariculture Use | South Africa | Marine | phytoplankton abundance and community composition, HABs | South African Water Quality Guidelines for Coastal Marine Waters - Natural Environment and Mariculture Use includes plankton as variables for assimilating, distributing and sequestrating nutrients (phosphate, nitrate, nitrite, silicon), metals (arsenic, cadmium, copper, nickel), and toxic substances (chlorine produced oxidants, sulphide, DDT) in the water column. Phytoplankton properties (e.g., toxins, community composition and abundance) are also used for monitoring the status of light penetration, and the concentrations of dissolved oxygen and algal toxins.  *Reference: Republic of South Africa, Department of Forestry, Fisheries and the Environment. 2022. South African Water Quality Guidelines for Coastal Marine Waters - Natural Environment and Mariculture Use. Cape Town. 162pp,* [*https://www.dffe.gov.za/sites/default/files/legislation/2023-09/waterqualityguideline2022.pdf*](https://www.dffe.gov.za/sites/default/files/legislation/2023-09/waterqualityguideline2022.pdf) |
| 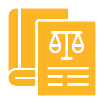 | UK Marine Strategy | UK | Marine | plankton biodiversity, abundance, biomass and community structure | The UK Marine Strategy aims to achieve Good Environmental Status in the UK seas through sustainable management. It is similar to the EU Marine Strategy Framework Directive. It requires the assessment of plankton biodiversity variables against targets representing Good Environmental Status for pelagic habitats. Plankton is measured under description one (biodiversity), four (food webs) and five (eutrophication). The UK Marine Strategy follow the OSPAR thresholds for the plankton variables.  *Reference: HM Government, 2011. UK Marine Policy Statement, 51pp,* [*https://www.gov.uk/government/publications/uk-marine-policy-statement*](https://www.gov.uk/government/publications/uk-marine-policy-statement) |
| 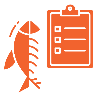 | Commercial copepod trawling licenses with total allowable catch (TAC) | Norway | Marine | *Calanus finmarchicus* catch | Under the Norwegian regulation for harvesting *Calanus finmarchicus* for 2024 Norway allow for Norwegian vessels to harvested and land a total of 254000 tons of copepods each year within certain parts of the EEZ and in international waters. 3000 tons are reserved for experimental and research purposes.  *Reference: Nærings- og fiskeridepartementet. Forskrift om regulering av høsting av rødåte i 2024 (Commercial copepod trawling licences with total allowable catch),* [*https://lovdata.no/dokument/LTI/forskrift/2023-12-13-2032*](https://lovdata.no/dokument/LTI/forskrift/2023-12-13-2032) |
| 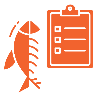 | CCAMLR Conservation measures | Southern Ocean/  Antarctica | Marine | krill catch | The main goal of the CCAMLR Conservation Measures is to ensure the sustainable management and preservation of marine living resources in the Antarctic region through a science-based approach. CCAMLR's regulations for krill focus on establishing catch limits and spatial management zones to prevent overfishing and ensure that krill populations remain healthy, as they are crucial for the ecological balance of the Southern Ocean's food web.  *Reference: Convention on the Conservation of Antarctic Marine Living Resources. 2023. Schedule of Conservation Measures 2023/24,* [*https://www.ccamlr.org/en/system/files/e-schedule2023-24.pdf*](https://www.ccamlr.org/en/system/files/e-schedule2023-24.pdf) |
| 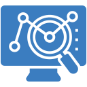 | The River Uruguay Administrative Commission, Periodic Beach Surveillance | Argentina and Uruguay | Freshwater | cyanobacteria abundance and algal blooms, algal toxins (*cylindrospermopsin* and *microcystins*) | The 'Uruguay River Beach Monitoring Programme' was initiated in 2007 by the Administrative Commission of the Uruguay River (CARU) to monitor the water quality of the river Uruguay at the stretch shared between Argentina and Uruguay. The technical teams from both commissions conduct in situ measurements and collect water samples for analysis in public and private laboratories of both countries. CARU provides interested parties with information about the sanitary condition of the beach water. This information is generated by comparing the results of cyanobacteria abundance and algal blooms in water samples from the river with reference values for recreational water use.  *Reference: River Uruguay Administrative Commission. Programa de Vigilancia de Playas del Rio Uruguay ‘C.A.R.U. – Comisión Administradora del Río Uruguay’ (The Periodic Beach Surveillance),* [*https://www.caru.org.uy/web/2017/12/programa-de-vigilancia-de-playas-del-rio-uruguay/*](https://www.caru.org.uy/web/2017/12/programa-de-vigilancia-de-playas-del-rio-uruguay/) |
| 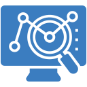 | National Aquatic Resource Surveys | USA | Freshwater and Marine | Chlorophyll *a*, phytoplankton and zooplankton assemblage, cyanobacteria, cyanotoxins (microcystin), enterocci | The National Aquatic Resource Surveys (NARS) are collaborative programmes between EPA, states, and tribes that aim to evaluate the condition of the nation’s coastal waters, lakes and reservoirs, rivers and streams, and wetlands through a statistical survey design. These surveys deliver essential, innovative, and uniformly consistent data on the quality of the nation’s aquatic environments. The plankton variables are under the Biological and Recreational descriptors.  *Reference: Reference: U.S. Environmental Protection Agency, National Aquatic Resource Surveys* [*https://www.epa.gov/national-aquatic-resource-surveys*](https://www.epa.gov/national-aquatic-resource-surveys) |
| 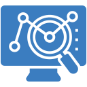 | Long-Term Ecological Research Program (LTER) | Brazil | Freshwater and Marine | primary production, bacterioplankton, phytoplankton, zooplankton (including ichyoplankton) | The Long-Term Ecological Research Programme (LTER), has been a pioneering initiative and strategic vision of the Federal Government since 1999. The LTER Programme finances a network of reference sites for scientific research in Brazil on Ecosystem Ecology. The data produced in the LTER Programme feeds the Brazilian Biodiversity Information System (SiBBr), which is part of the Global Biodiversity Information Facility (GBIF), a multilateral initiative of approximately 60 participating countries. SiBBr generates and makes available knowledge, providing subsidies for government management related to conservation and sustainable use. For example, the National Environmental Policy (Law No. 6.938) and the National Climate Change Policy (Law No. 12.187).  *Reference: Cordeiro et al. 2022, Long-term monitoring projects of Brazilian marine and coastal ecosystems, PeerJ 10:e14313* [*https://doi.org/10.7717/peerj.14313*](https://doi.org/10.7717/peerj.14313) |
| 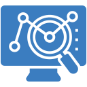 | NOAA Integrated Ecosystem Assessment (IEA) programme | USA | Freshwater and Marine | Chlorophyll *a*, abundance and biomass of phyto- and zooplankton, plankton community composition and size structure, HABs, seasonal biovolume | The NOAA Integrated Ecosystem Assessment (IEA) programme is a NOAA-wide initiative that oversees the direction and execution of Integrated Ecosystem Assessments. It is an approach to Ecosystem-Based Management that integrates all components of an ecosystem, including human needs and activities, into the decision-making process. The programme includes various plankton variables for which their monitoring status varies per state or/and region (e.g., Gulf of Alaska, Gulf of Mexico).  *Reference: NOAA Integrated Ecosystem Assessment (IEA) program,* [*https://www.integratedecosystemassessment.noaa.gov/about-iea*](https://www.integratedecosystemassessment.noaa.gov/about-iea) |
| 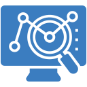 | Plan provincial de prevención y control de marea roja provincial (Plan for Prevention and Control of Red Tide in the Maritime Coast of the Chubut Province) | Argentina | Marine | Harmful phytoplankton species | In 2006, the Province of Chubut (Argentina) implemented the Plan provincial de prevención y control de marea roja provincial (Provincial Plan for Red Tide Prevention and Control) along the Maritime Coast of Chubut Province, under Decree No. 309/11. This programme involves the collection of phytoplankton, water, bivalve mollusk, and gastropod samples to monitor harmful phytoplankton species and related environmental parameters, as well as to control mollusk toxicity. Additionally, it encompasses education and dissemination about the red tide phenomenon, as well as symptom detection in hospitals and healthcare centres across the province. The programme also enforces bans on mollusk extraction and trade within the province.  *Reference: Province of Chubut, Plan provincial de prevención y control de marea roja provincial,* [*http://marearoja.chubut.gov.ar/*](http://marearoja.chubut.gov.ar/) |
| 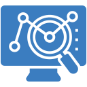 | Research Network of Marine-Coastal Stressors in Latin America and the Caribbean (REMARCO) | Argentina, Belice, Brazil, Chile, Colombia, Costa Rica, Cuba, Ecuador, El Salvador, Guatemala, Honduras, Mexico, Nicaragua, Panama, Peru, Dominican Republic, Uruguay, Venezuela | Marine | Abundance and biodiversity of harmful microalgae | REMARCO “Research Network of Marine-Coastal Stressors in Latin America and the Caribbean” is an interdisciplinary research network that aims to address environmental problems of the marine-coastal ecosystems of Latin America and the Caribbean. It generates validated information on chemical and microplastic pollution, ocean acidification, and harmful algal blooms to inform decision-makers and communities about accelerated environmental changes.  *Reference: REMARCO official webpage:* [*https://remarco.org/en/REMARCO/*](https://remarco.org/en/REMARCO/) |
| 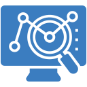 | Reef 2050 Integrated Monitoring and Reporting Programme | Australia | Marine | Chlorophyll a, abundance and diversity of bacteria, phytoplankton, zooplankton. Effect of warming on the Community Temperature Index for Archaea, Bacteria, phytoplankton and zooplankton | The Reef 2050 Integrated Monitoring and Reporting Programme is a collaborative initiative by the Australian and Queensland governments to monitor the status of the Great Barrier Reef through comprehensive data integration and reporting. It combines scientific research, traditional knowledge, and stakeholder input to support adaptive management, conservation efforts and policy decisions for the reef's long-term sustainability.  *Reference: Great Barrier Reef Marine Park Authority 2024. Great Barrier Reef Outlook Report 2024,* [*https://outlookreport.gbrmpa.gov.au/*](https://outlookreport.gbrmpa.gov.au/) |
| 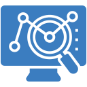 | Trilateral Monitoring and Assessment Programme (TMAP) | Netherlands, Germany, Denmark | Marine | Chlorophyll *a* as proxy for phytoplankton biomass | Joint monitoring programme of the Wadden Sea States within the framework of the Trilateral Wadden Sea Cooperation (TWSC). Chlorophyll *a* is used as a proxy to assess eutrophication in coordination with the EU Water Framework Directive (WFD).  *Reference: Wadden Sea Quality Status Report,* [*https://qsr.waddensea-worldheritage.org/*](https://qsr.waddensea-worldheritage.org/) |
| 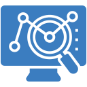 | South African Shellfish Monitoring and Control Programme | South Africa | Marine | Phytoplankton species identification, distribution, biotoxins | The South African Shellfish Monitoring and Control Programme is a regulatory system that ensures the safety and quality of shellfish by monitoring contaminants (e.g., biotoxins, heavy metals, and microbial pathogens), and aims to support the industry's sustainability and protect public health. Phytoplankton is measured for monitoring biotoxins in the water column and shellfish.  *Reference: Republic of South Africa, Department of Environment, Forestry and Fisheries. 2021. South African Shellfish Monitoring and Control Programme, 65pp.,* [*https://www.dffe.gov.za/sites/default/files/legislations/programmemanual_shellfish_monitoringcontrol.pdf*](https://www.dffe.gov.za/sites/default/files/legislations/programmemanual_shellfish_monitoringcontrol.pdf) |
